# Addendum: Heliconical smectic phases formed by achiral molecules

**DOI:** 10.1038/s41467-018-05334-x

**Published:** 2018-07-17

**Authors:** Jordan P. Abberley, Ross Killah, Rebecca Walker, John M. D. Storey, Corrie T. Imrie, Mirosław Salamończyk, Chenhui Zhu, Ewa Gorecka, Damian Pociecha

**Affiliations:** 1 Department of Chemistry, King’s College, University of Aberdeen, Aberdeen, AB24 3UE UK; 2 Advanced Light Source, Lawrence Berkeley National Laboratory, 1 Cyclotron Road, Berkeley, CA 94720 USA; 3 Department of Physics and Liquid Crystal Institute, Kent State University, 1425 Lefton Esplanade, Kent, OH 44242 USA; 4 Faculty of Chemistry, University of Warsaw, Zwirki i Wigury 101, Warsaw, 02-089 Poland

**Keywords:** Structure of solids and liquids, Molecular self-assembly, Liquid crystals

Addendum to: *Nature Communications* 10.1038/s41467-017-02626-6; published online 15 Jan 2018

We would like to make our readers aware of the related publications by S.P. Sreenilayam et al. (*Nat. Commun*. **7**, 11369 (2016) and *Phys. Rev. Mat*. **1**, 035604 (2017)), which report the spontaneous helix formation in a polar smectic liquid crystal phase made of achiral bent-core mesogens.

