# Supplementary material for: Heliconical smectic phases formed by achiral molecules
Source: Nat Commun. 2018 Jan 15;9:228. doi: 10.1038/s41467-017-02626-6 (PMC5768771; doi:10.1038/s41467-017-02626-6)
Supplement: Supplementary file 2 — Supplementary Information [file 41467_2017_2626_MOESM2_ESM.pdf]

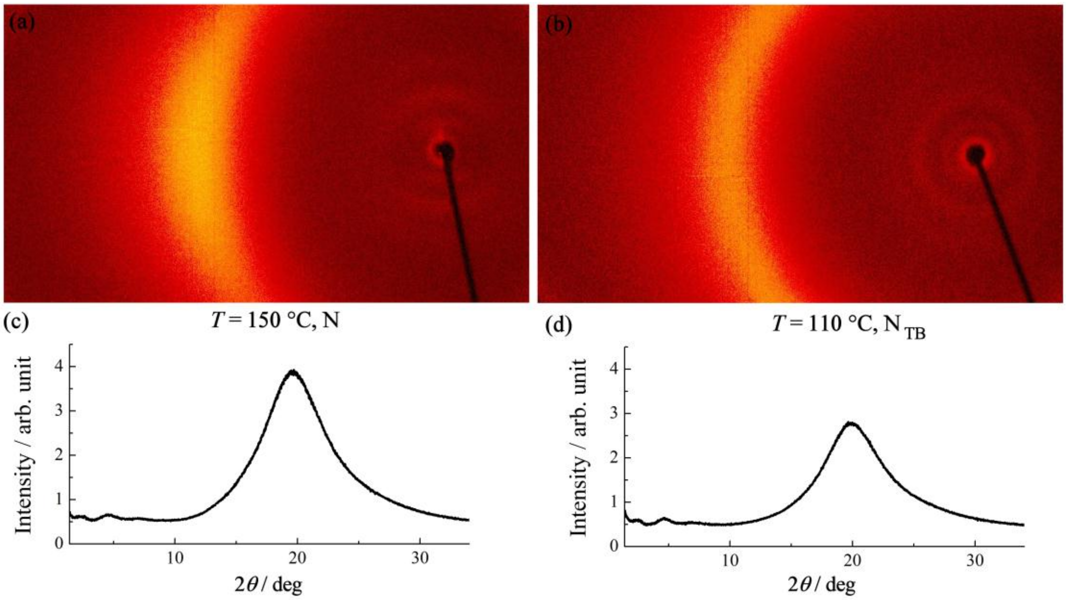

Supplementary Figure 1. X-ray diffraction in N and N_{TB} phases
Wide angle 2D XRD patterns obtained for nematic (a) and twist-bend nematic (b) phases for homologue *n*=5, and the corresponding dependence of the diffracted intensity vs. diffraction angle (c) and (d).

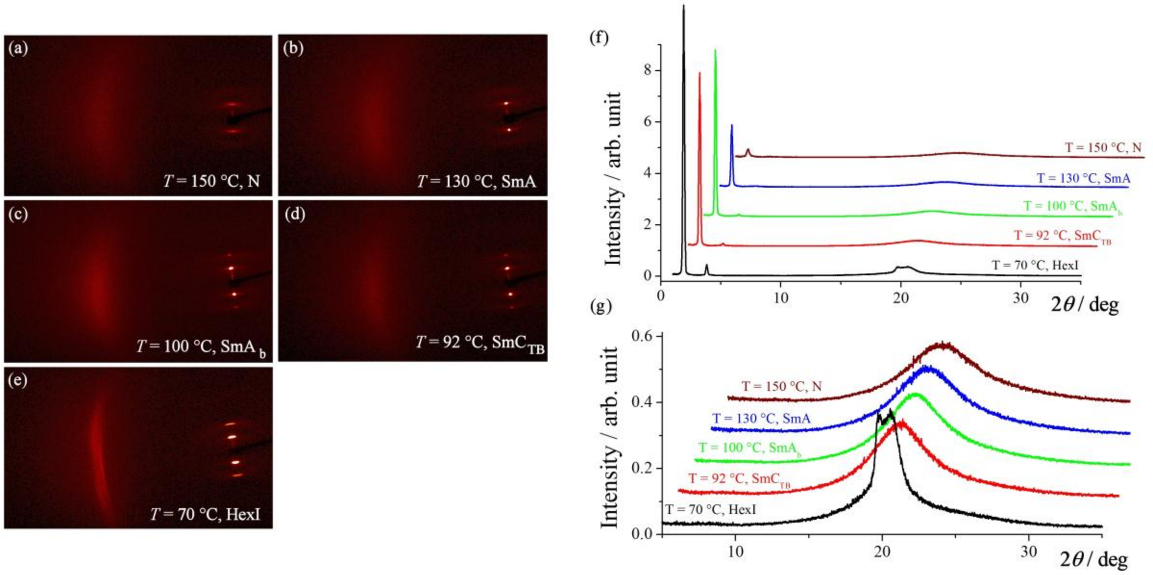

Supplementary Figure 2. X-ray diffraction in smectic phases
Wide angle 2D XRD patterns obtained for an aligned sample of compound *n*=8 in nematic (a), smectic A (b), smectic A_b (c), smecic C_{TB} (d) and hexatic I (e) phases. (f) Integrated intensity of diffraction signals vs. diffraction angle 2θ, in (g) magnification of the high angle range of the integrated diffractograms.

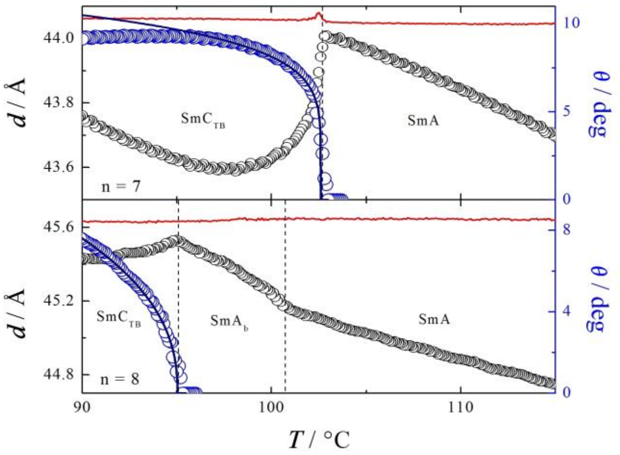

Supplementary Figure 3. Layer spacing and tilt angle

Temperature evolution of the layer thickness (black circles) near the transition between the orthogonal and tilted smectic phases for homologues *n*=7 and *n*=8. For homologue *n*=7, the SmA-SmC$_{TB}$ transition is weakly first order, as evidenced by the appearance of a two-phase region manifested as a slight increase in the width of the diffraction signal related to the layer periodicity (red line), whereas the SmA$_b$-SmC$_{TB}$ phase transition in homologue *n*=8 is continuous. Temperature dependence of tilt angle (blue circles), obtained from the decrease of the layer spacing in the SmC$_{TB}$ phase with respect to the layer thickness value extrapolated from the data recorded in the orthogonal phase, has been fitted to a power law with critical exponent β equal to 0.15 and 0.40 for compounds *n*=7 and *n*=8, respectively.

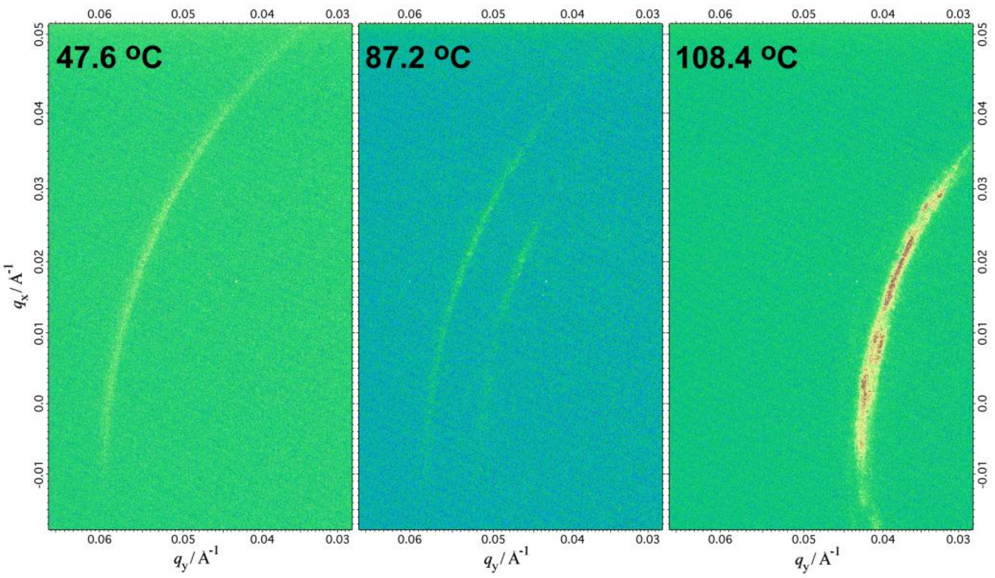

Supplementary Figure 4. Resonant X-ray diffraction

2D resonant soft X-ray diffraction patterns measured for homologue *n*=7 in the HexI (47.6 °C) and SmC$_{TB}$ (108.4 °C) phases, and in the phase coexistence range (87.2 °C).

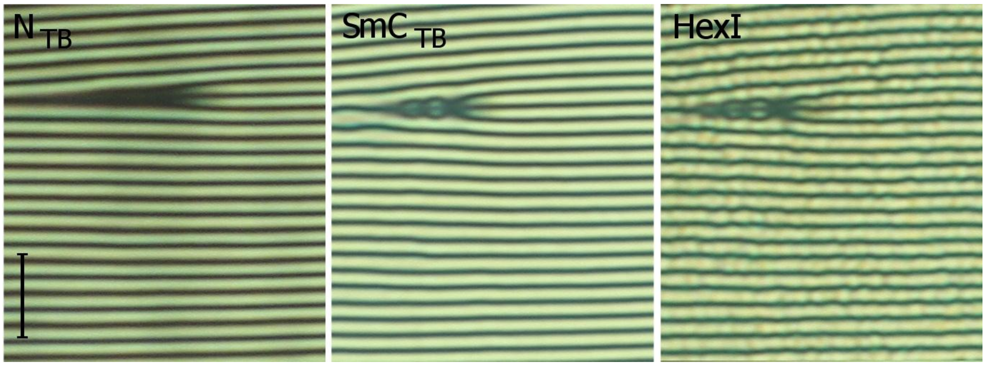

Supplementary Figure 5. Optical texture in planar cell
Evolution of the stripe texture through the phase sequence N_{TB} – SmC_{TB} – HexI observed in the 50-50 wt.% mixture of homologues *n*=6 and *n*=7 in 1.9 micron thick cell with planar anchoring. Scale bar corresponds to 10 μm.

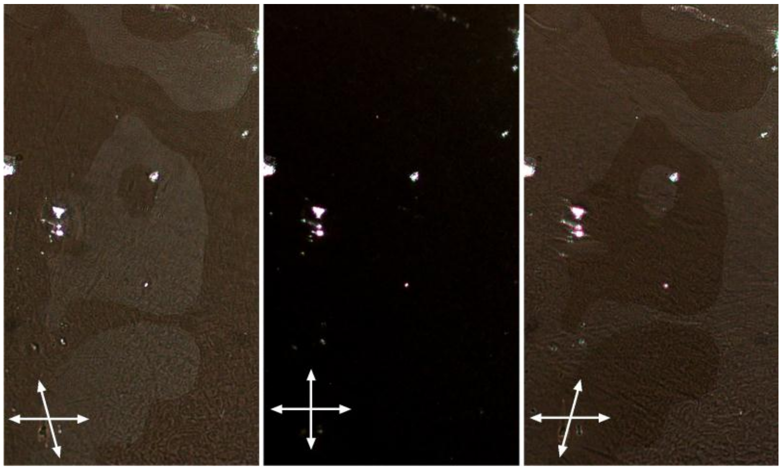

Supplementary Figure 6. Optical texture in hometropic cell
Domains with optical activity observed in HexI phase of homologue *n*=7 by slight de-crossing of polarizers (image brightness and contrast were artificially increased to better visualize the domains).

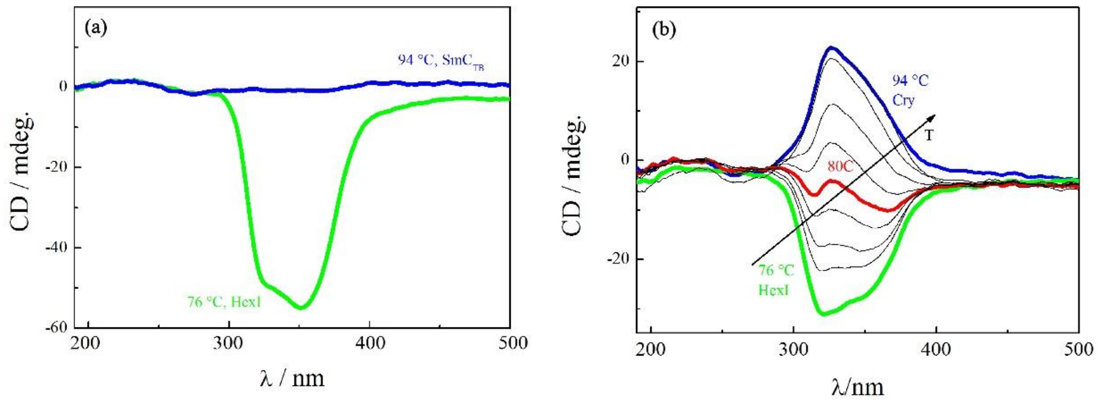

Supplementary Figure 7. Circular dichroism studies
CD signal recorded for homologue *n*=7: (a) in the HexI and SmC$_{TB}$ phases and (b) upon the re-crystallization of the sample slowly heated from HexI phase.

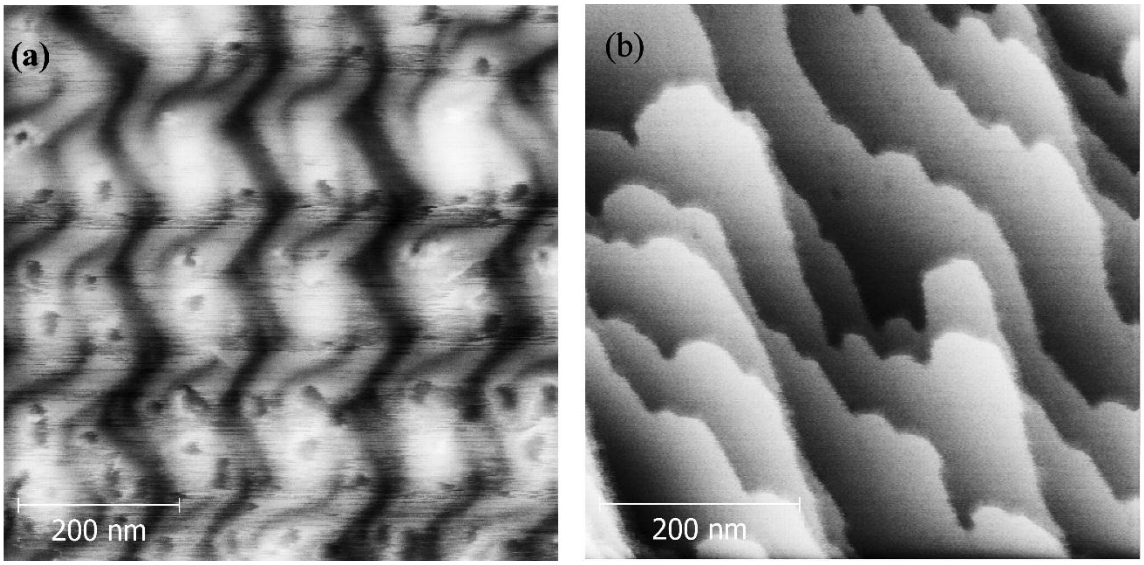

Supplementary Figure 8. AFM studies
(a) AFM image of filaments formed in the HexI phase. (b) Re-crystallization of the sample is accompanied by the change of morphology to flat plates. Images were taken for homologues (a) *n*=7 and (b) *n*=5 at room temperature.

Supplementary Discussion

X-ray studies

Both nematic phases, N and N_{TB}, showed similar X-ray patterns (Supplementary Figure 1). In the low angle region very weak diffraction signals were visible, corresponding to the full (*L*) and half molecular lengths (*L*/2). However, upon transition to the N_{TB} phase the partial alignment of the sample present in the N phase was lost.

For homologues with *n*>6, exhibiting the SmA phase below the N phase, the width of the low-angle signal corresponding to full molecular length continuously decreased on cooling, indicating that the system has a tendency to lock to the lamellar structure with $d = L$. X-ray diffraction measurements performed for compound *n*=8, with a sequence of 4 smectic phases below the nematic phase, revealed that three of the phases assigned as: SmA, SmA_b and SmC_{TB}, have no long-range positional order within the smectic layers (liquid-like smectic phases). The transition to the lowest temperature lamellar phase, HexI, is marked by a sudden change in the high angle range of the X-ray pattern, the diffuse signal observed in the SmA, SmA_b, and SmC_{TB} phases, narrows and becomes doubled (Supplementary Figure 2). The equatorial position of one of the high angle signals, indexed as (020), unambiguously determines that the tilt direction is toward nearest neighbour molecules in the in-plane hexagonal lattice. This confirms the HexI phase assignment.

The evolution of the layer spacing at the SmA-SmC_{TB} phase transition points toward the weakly first order transition for homologue *n*=7, as evidenced by a slight increase of the width of the diffraction signal caused by a two phase co-existence over a ~0.5 K temperature range close to the transition temperature, and a continuous transition for homologue *n*=8 (Supplementary Figure 3). The tilt angle calculated from the layer thickness decreases, following critical behaviour $\theta=\theta_0((T_c-T)/T_c)^{\beta}$ with critical exponent β~0.15 and 0.40 for compounds *n*=7 and *n*=8, respectively.

The resonant X-ray diffraction studies clearly revealed the signal arising from the superstructure in the HexI and SmC_{TB} phases (Supplementary Figure 4). The small temperature range (2-3 K) in which both signals coexists indicates a first order transition.

Optical and AFM studies

In both the N_{TB} and SmC_{TB}, phases, in the cells with planar anchoring the characteristic stripe texture was found, the periodicity of the stripes was defined by the cell thickness. Moreover, by studying the mixture of homologues *n*=6 and n=7 it was confirmed that the stripe pattern is not affected by the N_{TB} - SmC_{TB} phase transition (Supplementary Figure 5), it persists also in HexI phase however the strips start to undulate.

The optical texture of the HexI phase, in the cell with homeotropic anchoring and observed with slightly de-crossed polarizers, revealed the presence of domains with small optical activity (Supplementary Figure 6). The origin of the optical activity is unclear. It may be due to the lowering of the local symmetry by molecular tilting combined with local biaxial order (so called 'layer chirality' [1]) or due to the formation of chiral excimers consisting of twisted molecular pairs [2].

The chiral nature of the HexI phase is consistent with the observation of a CD signal at the wavelength of the optical absorption band of the material, 350 nm (Supplementary Figure 7); the absorption at this wavelength is due to the presence of the imine unit in the molecular structure. On heating, the CD signal decreases and disappears at the transition

to SmC_{TB} (*n*=7), to SmA phase (*n*=10) or to N_{TB} phase (*n*=6). Lack of CD signal in N_{TB} and SmC_{TB} phases, indicates that either chiral domains in these phases are smaller than the wavelength of visible light which averages the chiral effects to zero or the biaxality that is required for 'layer chirality' is zero or very small. Interestingly, for material *n*=7 in some heating runs the CD signals reverses its sign upon the sample re-crystallization (HexI phase in this material is metastable). The non-zero CD signal suggests that also crystalline phase has chiral structure, however this should be taken with some caution as the CD data can be unambiguously analysed only for non birefringent samples.

Using AFM revealed the entangled helical filament morphology in HexI phase at room temperature for rapidly cooled samples (the procedure preserved the HexI phase in low temperature) (Supplementary Figure 8a). On sample recrystallization the morphology changed and plate-like domains are formed (Supplementary Figure 8b).

Supplementary Methods

Materials

All materials were used as purchased (Sigma Aldrich, Alfa Aesar), unless otherwise stated. Dichloromethane was dried over 3Å molecular sieves prior to use. Solvents were evaporated at approximately 20 mm Hg using a water aspirator pump connected to a Buchi rotary evaporator. Trace solvents were removed from solids in a Thermo Scientific vacuum oven at 1.0 mm Hg and 50 °C. Reactions were monitored using thin layer chromatography (TLC) carried out on aluminium-backed plates with a coating of Merck Kieselgel 60 F254 silica and an appropriate solvent system. Silica gel coated aluminium plates were purchased from Merck KGaA. Spots were visualised using UV light (254 nm) or by oxidation with either an aqueous permanganate dip or iodine.

Organic Synthesis

Synthesis of 6-bromo-1-[4-(4-bromophenyl)phenyl]hexan-1-one (BrBK5Br).

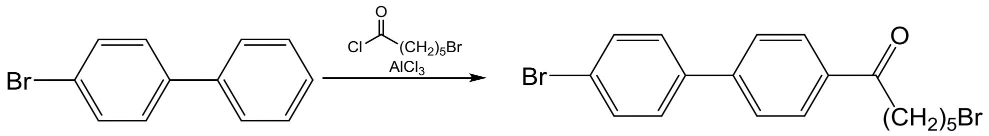

Supplementary Figure 9: Synthesis of **BrBK5Br**.

The synthesis of 6-bromo-1-[4-(4-bromophenyl)phenyl]hexan-1-one (**BrBK5Br**) was performed according to Supplementary Figure 9 [3]. Significantly greater yields were noted for this reaction when all glassware was wrapped in aluminium foil, and this is presumed to be due to photolytic debromination of the reagent bromobiphenyl *via* a free radical pathway [4]. A stirred mixture of anhydrous aluminium chloride (12.503 g, 9.38 x 10^{-2} mol, 1.2 mol. eq.) in dichloromethane (200 ml) was cooled to 0 °C, and to this a solution of 4-bromobiphenyl (18.26g, 7.83 x 10^{-2} mol, 1 mol. eq.) and 6-bromohexanoyl chloride (20.00 g, 9.36 x 10^{-2} mol, 1.2 mol. eq.) in dichloromethane (50 ml) was added dropwise in the absence of light, over a period of 30 min. After 1 h, the ice was removed and the mixture allowed to reach room temperature with stirring for a further 4 h. The solution was slowly added to a mixture of crushed ice (100 g), distilled water (100 ml) and 20 % hydrochloric acid (100 ml) to destroy the aluminium chloride complex. The organic layer was collected and washed with distilled water (2 x 200 ml), and dried over magnesium sulfate. *In vacuo* removal of the dichloromethane gave an off-white solid which was recrystallised twice from boiling ethanol (200 ml x 2) and dried in a vacuum oven at 50 °C overnight.

***BrBK5Br** - 6-bromo-1-[4-(4-bromophenyl)phenyl]hexan-1-one:*
Yield: 26.52 g, 82.9 %.
Melting point: 81.5 – 82.2 °C.
Infrared $\bar{\nu}$ cm^{-1}: 2952, 2866 (sp_3 C-H); 1684 (C=O); 1604 (Ar C=C); 807 (p. disubs. benzene C-H), 665 (C-Br).
^1H NMR: (300 MHz, Chloroform-d) δ ppm: 1.50 – 1.66 (m, 2 H, -C(O)-CH_2-CH_2-**CH_2**-CH_2-CH_2-Br), 1.75 - 1.88 (m, 2 H, -C(O)-CH_2-**CH_2**-CH_2-),

1.90 – 2.02 (m, 2 H, -CH$_2$-**CH$_2$**-CH$_2$-Br), 3.05 (t, J=7.3 Hz, 2 H, -C(O)-**CH$_2$**-CH$_2$-), 3.47 (t , J=6.7 Hz, 2 H, -CH$_2$-**CH$_2$**-Br), 7.48 – 7.55 (m, 2 H, Ar), 7.59 – 7.70 (m, 4 H, Ar), 8.02 – 8.08 (m, 2 H, Ar).
^{13}C NMR: (75 MHz, Chloroform-d) δ ppm: 23.38, 27.89, 32.63, 33.56, 38.35, 122.65, 127.05, 128.72, 128.80, 132.11, 135.99, 138.80, 144.39, 199.40.

Synthesis of 1-bromo-4-[4-(6-bromohexyl)phenyl]benzene (BrB6Br).

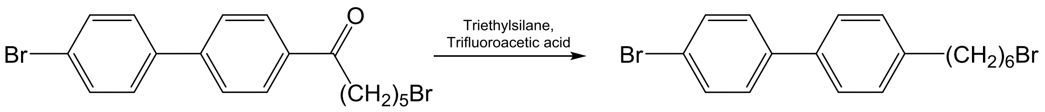

Supplementary Figure 10: Synthesis of **BrB65Br**

The synthesis of 1-bromo-4-[4-(6-bromohexyl)phenyl]benzene (**BrB6Br**) was performed according to Supplementary Figure 10, as previously reported by Jansze et al. [5]. Thus, a dry solution of BrBK5Br (26.508 g, 6.47 x 10^{-2} mol, 1 mol. eq.) in trifluoroacetic acid (60.0 ml, 3.53 x 10^{-1} mol, 5.5 mol. eq.) was cooled to 0 °C, and triethylsilane (23.0 ml, 1.44 x 10^{-1} mol, 2.2 mol. eq.) added dropwise over a period of 20 min. After 30 min, dry dichloromethane (40 ml) was added, and left to stir for 1 h under ice and then overnight at room temperature. The product mixture was poured over a mixture of dichloromethane (200 ml) and distilled water (300 ml), and the organic layer separated. The aqueous layer was washed twice further with dichloromethane (100 ml x 2), and the organic layers recombined and dried over magnesium sulfate. After the *in vacuo* removal of the dichloromethane, the pale yellow crystals were crushed into a fine powder and left to stir overnight under high vacuum at 40 °C to remove the hexaethyldisiloxane by-product. Recrystallising twice from boiling ethanol (2 x 150 ml) and drying in a vacuum oven overnight at 50 °C afforded the product as a bright white powder.

***BrB6Br** - 1-bromo-4-[4-(6-bromohexyl)phenyl]benzene*:
Yield: 20.13 g, 78.5 %.
Melting point: 74.9 – 75.2 °C.
Infrared $\bar{\nu}$ cm-1: 2932, 2856 (sp3 C-H); 1607, 1480 (Ar C=C); 804 (p. disubs. benzene C-H), 647 (C-Br).
^{1}H NMR: (300 MHz, Chloroform-d) δ ppm: 1.33 – 1.55 (m, 4 H, -CH$_2$-**CH$_2$**-**CH$_2$**-CH$_2$-CH$_2$-Br), 1.61 - 1.75 (m, 2 H, Ar-CH$_2$-**CH$_2$**-CH$_2$-), 1.89 (quin, J=7.1 Hz, 2 H, -CH$_2$-**CH$_2$**-CH$_2$-Br), 2.62 – 2.77 (m, 2 H, Ar-**CH$_2$**-CH$_2$-), 3.43 (t , J=6.8 Hz, 2 H, -CH$_2$-**CH$_2$**-Br), 7.21 – 7.30 (m, 2 H, Ar), 7.41 – 7.53 (m, 4 H, Ar), 7.53 – 7.61 (m, 2 H, Ar).
^{13}C NMR: (75 MHz, Chloroform-d) δ ppm: 28.02, 28.39, 31.18, 32.73, 33.90, 35.43, 121.21, 126.83, 128.56, 128.95, 131.81, 137.42, 140.04, 142.18.

Synthesis of 4-{[6-(4-(4-bromophenyl)phenyl)hexyl]oxy}-benzaldehyde (BrB6OPhK).

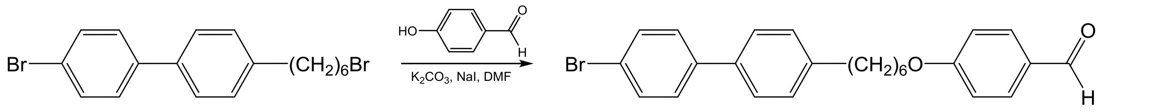

Supplementary Figure 11: Synthesis of 4-{[6-(4-(4-bromophenyl)phenyl)hexyl]oxy} benzaldehyde (**BrB6OPhK**).

The synthesis of 4-{[6-(4-(4-bromophenyl)phenyl)hexyl]oxy}benzaldehyde was carried out using Williamson's ether synthesis catalysed *via* an in-situ Finkelstein substitution, as outlined in Supplementary Figure 11. Thus, to a stirring solution of 1-bromo-4-[4-(6-bromohexyl)phenyl]benzene (6.083 g, 1.54 x 10^{-2} mol, 1.0 mol. eq.) in N,N'-dimethylformamide (30 ml), 4-hydroxybenzaldehyde (1.887 g, 1.55 x 10^{-2} mol, 1.0 mol. eq.), potassium carbonate (21.294 g, 1.54 x 10^{-1} mol, 10.0 mol. eq.) and sodium iodide (207 mg, 1.38 x 10^{-3} mol, 0.1 mol. eq.) was added. The mixture was stirred at 90 °C for 24 h, then left to cool to room temperature before adding to distilled water (350 ml). The resulting cream paste-like precipitate was collected *via* vacuum filtration, added to dichloromethane (50 ml), and washed twice with distilled water (50 ml x 2). The organic layer was dried over magnesium sulfate, and the solvent removed *via* rotary evaporation to yield off-white powder. The powder was twice recrystallised from ethanol (30 ml x 2) and dried *in vacuo* at 50 °C to give the title compound.

***BrB6OPhK** - 4-{[6-(4-(4-bromophenyl)phenyl)hexyl]oxy}benzaldehyde:*
Yield: 5.591 g, 83.1 %.
Melting point: 87.7 – 89.3 °C.
Infrared $\bar{\nu}$ cm^{-1}: 2934, 2859 (sp^3 C-H); 1691 (C=O); 1600, 1577, 1477 (Ar C=C); 806 (p. disubs. benzene C-H).
^{1}H NMR: (300 MHz, Chloroform-d) δ ppm: 1.39 – 1.62 (m, 4 H, -CH_2-**CH_2**-**CH_2**-CH_2-CH_2-O-), 1.71 (quin, J=7.5 Hz, 2 H, Ar-CH_2-**CH_2**-CH_2-), 1.78 - 1.94 (m, 2 H, -CH_2-**CH_2**-CH_2-O-), 2.69 (t, J=7.7 Hz, 2 H, Ar-**CH_2**-CH_2-), 4.06 (t , J=6.40 Hz, 2 H, -CH_2-**CH_2**-O-), 7.00 (d, J=8.7 Hz, 2 H, Ar), 7.24 – 7.31 (m, 2 H, Ar), 7.42 – 7.52 (m, 4 H, Ar), 7.53 – 7.62 (m, 2 H, Ar), 7.81 – 7.90 (m, 2 H, Ar), 9.90 (s, 1 H, **HC(O)**-Ar).
^{13}C NMR: (75 MHz, Chloroform-d) δ ppm: 25.86, 28.93, 28.98, 31.27, 35.46, 68.32, 114.75, 121.23, 126.81, 128.54, 128.97, 129.81, 131.82, 131.99, 137.41, 140.01, 142.22, 164.22, 190.78

Synthesis of 4-{4-[6-(4-formylphenoxy)hexyl]phenyl}benzonitrile (CB6OPhK).

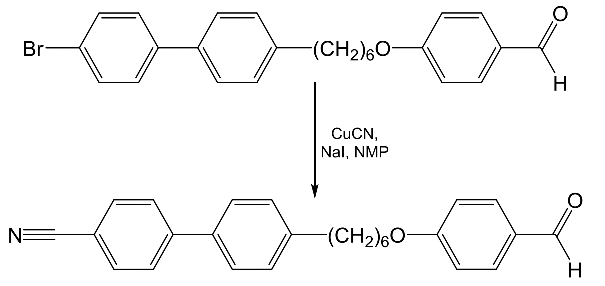

Supplementary Figure 12: Synthesis of 4-{4-[6-(4-formylphenoxy)hexyl]phenyl} benzonitrile (**CB6OPhK**).

A Rosenmund - von Braun reaction was used in the cyanation of 4-{[6-(4-(4-bromophenyl)phenyl)hexyl]oxy}benzaldehyde, as shown in Supplementary Figure 12 [6]. Copper (I) cyanide (2.230 g, 2.49 x 10^{-2} mol, 2.0 mol. eq.), 4-{[6-(4-(4-bromophenyl)phenyl)hexyl]oxybenzaldehyde (5.407 g, 1.24 x 10^{-2} mol, 1.0 mol. eq.), and sodium iodide (1.829 g, 1.22 x 10^{-2} mol, 1.0 mol. eq.) were refluxed in N-methyl-2-pyrollidone (50 ml) at 200 °C for 5 h with rapid stirring. The black solution was then cooled to room temperature, and 9 % ammonium hydroxide solution (20 ml) was added, and the mixture stirred for 10 min while bubbling any product gases through a 33% solution of sodium hypochlorite. The dichloromethane layer was collected and washed with brine (100 ml x 2), then with 2M hydrochloric acid (100 ml x 2), and finally with distilled water (100 ml x 2). The organic layer was collected and dried over magnesium sulfate, and the solvent removed using rotary evaporation to give a dark brown liquid. The crude product was purified *via* silica gel chromatography using dichloromethane as an eluent, then twice recrystallised from ethanol (30 ml x 2) and dried *in vacuo* at 50 °C overnight.

***CB6OPhK** - 4-{4-[6-(4-formylphenoxy)hexyl]phenyl}benzonitrile:*
Yield: 3.226 g, 67.8 %.
Melting point: 80.6 – 81.8 °C.
Infrared $\bar{\nu}$ cm^{-1}: 2928, 2858 (sp^3 C-H); 2223 (C≡N); 1689 (C=O); 1599, 1574, 1493 (Ar C=C); 812 (p. disubs. benzene C-H).
^1H NMR: (300 MHz, Chloroform-d) δ ppm: 1.39 – 1.62 (m, 4 H, -CH_2-**CH_2**-**CH_2**-CH_2-CH_2-O-), 1.72 (quin, J=7.5 Hz, 2 H, Ar-CH_2-**CH_2**-CH_2-), 1.79 - 1.93 (m, 2 H, -CH_2-**CH_2**-CH_2-O-), 2.71 (t, J=7.7 Hz, 2 H, Ar-**CH_2**-CH_2-), 4.06 (t , J=6.4 Hz, 2 H, -CH_2-**CH_2**-O-), 6.96 – 7.07 (m, 2 H, Ar), 7.31 (d, J=8.3 Hz, 2 H, Ar), 7.49 – 7.58 (m, 2 H, Ar), 7.66 – 7.78 (m, 4 H, Ar), 7.81 – 7.91 (m, 2 H, Ar), 9.90 (s, 1 H, **HC(O)**-Ar).
^{13}C NMR: (75 MHz, Chloroform-d) δ ppm: 25.86, 28.94, 28.98, 31.27, 35.51, 68.28, 110.58, 114.73, 119.06, 127.13, 127.48, 129.19, 129.77, 132.01, 132.59, 136.58, 143.45, 145.54, 164.19, 190.84.

Synthesis of 4-nitrophenyl-4-(alkoxy)benzoate esters (NxBeO*n*).

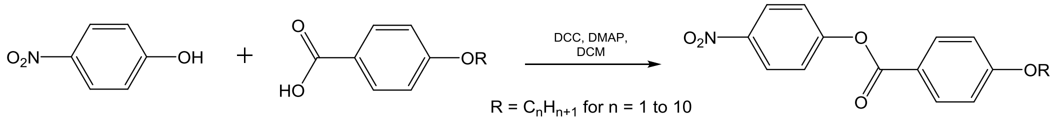

Supplementary Figure 13: Synthesis of 4-nitrophenyl-4-(alkoxy)benzoate esters.

4-Nitrophenyl-4-(alkyloxy)benzoate esters were synthesised according to Supplementary Figure 13 using a general Steglich ester synthesis as previously reported by Reddy *et al* [7]. As such, the required 4-alkyloxybenzoic acid (1.0 mol. eq.), 4-nitrophenol (1.0 mol. eq.), and dry dichloromethane (150 ml) were added to a conical flask and left to stir for 5 min. 4-Dimethylaminopyridine (0.10 mol. eq.) was added to give an instant yellow solution that was left to stir for 10 min, after which a solution of N,N'-dicyclohexylcarbodiimide (1.3 mol. eq.) in dry dichloromethane (50 ml) was added. Actual quantities used in the synthesis of all ten 4-nitrophenyl-4-(alkyloxy)benzoate intermediates are given in Table S1. The mixtures were left to stir for 12 h at room temperature, after which a white precipitate of N,N'-dicylohexyl urea formed. Most of the precipitate was initially removed *via* vacuum filtration, washing with copious dry dichloromethane, and the remaining through gravity filtration. The clear yellow filtrate was washed three times with a 5% potassium hydroxide solution (3 x 100 ml) and once with distilled water (150 ml), before the organic layer was collected and dried over magnesium sulfate. Dichloromethane was removed *via* rotary evaporation to yield a pale yellow crude solid that was recrystallised from boiling ethanol (150 ml) to give white crystals which were washed with copious ice-cold ethanol, and dried *in vacuo* for 12 h at 50 °C to give the reported yields.

Supplementary Table 1. Quantities of reagents used in the synthesis of 4-nitrophenyl-4-(alkoxy) benzoate esters

	**4-Alkoxybenzoic acid used:**	**Quantity of 4-Alkoxybenzoic acid used:**	**Quantity of 4-Nitrophenol used:**	**Quantity of DMAP used:**	**Quantity of DCC used:**
**n = 1**	4-Methoxybenzoic acid	5.009 g, 3.29 x 10^{-2} mol, 1.0 eq.	4.574 g, 3.29 x 10^{-2} mol, 1.0 eq.	437 mg, 3.58 x 10^{-3} mol, 0.11 eq.	8.872 g, 4.27 x 10^{-2} mol, 1.3 eq.
**n = 2**	4-Ethoxybenzoic acid	5.467 g, 3.29 x 10^{-2} mol, 1.0 eq.	4.690 g, 3.37 x 10^{-2} mol, 1.0 eq.	525 mg, 4.30 x 10^{-3} mol, 0.13 eq.	9.356 g, 4.53 x 10^{-2} mol, 1.4 eq.
**n = 3**	4-Propoxybenzoic acid	3.972 g, 2.20 x 10^{-2} mol, 1.0 eq.	3.333 g, 2.40 x 10^{-2} mol, 1.1 eq.	317 mg, 2.59 x 10^{-3} mol, 0.12 eq.	5.846 g, 2.84 x 10^{-2} mol, 1.3 eq.
**n = 4**	4-Butoxybenzoic acid	6.394 g, 3.29 x 10^{-2} mol, 1.0 eq.	4.634 g, 3.33 x 10^{-2} mol, 1.0 eq.	427 mg, 3.50 x 10^{-3} mol, 0.11 eq.	8.740 g, 4.24 x 10^{-2} mol, 1.3 eq.
**n = 5**	4-Pentyloxybenzoic acid	6.911 g, 3.32 x 10^{-2} mol, 1.0 eq.	4.710 g, 3.39 x 10^{-2} mol, 1.0 eq.	411 mg, 3.36 x 10^{-3} mol, 0.10 eq.	8.502 g, 4.12 x 10^{-2} mol, 1.2 eq.

**n = 6**	4-Hexyloxybenzoic acid	1.632 g, 7.34 x 10^{-3} mol, 1.0 eq.	1.140 g, 8.19 x 10^{-3} mol, 1.1 eq.	107 mg, 8.76 x 10^{-4} mol, 0.12 eq.	1.927 g, 9.34 x 10^{-3} mol, 1.3 eq.
**n = 7**	4-Heptyloxybenzoic acid	7.773 g, 3.29 x 10^{-2} mol, 1.0 eq.	4.586 g, 3.30 x 10^{-2} mol, 1.0 eq.	491 mg, 4.02 x 10^{-3} mol, 0.12 eq.	8.503 g, 4.12 x 10^{-3} mol, 1.3 eq.
**n = 8**	4-Octyloxybenzoic acid	4.993 g, 1.99 x 10^{-2} mol, 1.0 eq.	2.851 g, 2.04 x 10^{-2} mol, 1.0 eq.	316 mg, 2.59 x 10^{-3} mol, 0.13 eq.	5.420 g, 2.63 x 10^{-2} mol, 1.3 eq.
**n = 9**	4-Nonyloxybenzoic acid	4.270 g, 1.61 x 10^{-2} mol, 1.0 eq.	2.328 g, 1.67 x 10^{-2} mol, 1.0 eq.	224 mg, 1.83 x 10^{-3} mol, 0.11 eq.	4.291 g, 2.08 x 10^{-2} mol, 1.3 eq.
**n = 10**	4-Decyloxybenzoic acid	4.862 g, 1.75 x 10^{-2} mol, 1.0 eq.	2.741 g, 1.97 x 10^{-2} mol, 1.1 eq.	287 mg, 2.35 x 10^{-3} mol, 0.13 eq.	4.565 g, 2.21 x 10^{-2} mol, 1.3 eq.

***NxBeO1** – 4-Nitrophenyl-4-methoxybenzoate:*

Yield: 7.85 g, 87.3 %.
Melting point: 162.0 – 162.8 °C.
Infrared $\bar{\nu}$ cm^{-1}: 3081 (sp^2 C-H); 2988, 2944, 2847 (sp^3 C-H); 1730 (C=O); 1609, 1583, 1490 (Ar C=C); 1513 (NO_2); 849, 832 (p. disubs. benzene C-H). ^{1}H NMR: (300 MHz, Chloroform-d) δ ppm: 3.94 (s, 3 H, -Ar-O-$\underline{\mathbf{CH_3}}$), 7.03 (d, J=9.0 Hz, 2H, Ar), 7.42 (d, J=9.2 Hz, 2 H, Ar), 8.17 (d, J=9.0 Hz, 2 H, Ar), 8.33 (d, J=9.2 Hz, 2 H, Ar).
^{13}C NMR: (75 MHz, Chloroform-d) δ ppm: 55.61, 114.09, 120.72, 122.68, 125.23, 132.54, 145.26, 155.94, 163.92, 164.42.

***NxBeO2** – 4-Nitrophenyl-4-ethoxybenzoate:*

Yield: 8.64 g, 91.4 %.
Melting point: 124.6 – 125.7 °C.
Infrared $\bar{\nu}$ cm^{-1}: 3081 (sp^2 C-H); 2990 (sp^3 C-H); 1727 (C=O); 1610, 1581, 1491 (Ar C=C); 1515 (NO_2); 843, 831 (p. disubs. benzene C-H).
^{1}H NMR: (300 MHz, Chloroform-d) δ ppm: 1.49 (t, *J*=7.0 Hz, 3 H, -O-CH_2-$\underline{\mathbf{CH_3}}$), 4.16 (q, *J*=7.1 Hz, 2 H, -O-$\underline{\mathbf{CH_2}}$-$CH_3$), 7.01 (d, *J*=9.0 Hz, 2 H, Ar), 7.42 (d, *J*=9.0 Hz, 2 H, Ar), 8.15 (d, *J*=9.0 Hz, 2 H, Ar), 8.33 (d, *J*=9.0 Hz, 2 H, Ar).
^{13}C NMR: (75 MHz, Chloroform-d) δ ppm: 14.66, 63.95, 114.49, 120.45, 122.69, 125.23, 132.54 145.23, 155.97, 163.85, 163.96.

***NxBeO3** – 4-Nitrophenyl-4-propoxybenzoate:*

Yield: 5.33 g, 80.4 %.
Melting point: 63.2 – 63.8 °C.
Infrared $\bar{\nu}$ cm^{-1}: 3075 (sp^2 C-H); 2973 (sp^3 C-H); 1723 (C=O); 1607, 1592, 1493 (Ar C=C); 1514 (NO_2); 847 (p. disubs. benzene C-H).
^{1}H NMR: (300 MHz, Chloroform-d) δ ppm: 1.08 (t, *J*=7.4 Hz, 3 H, -CH_2-$\underline{\mathbf{CH_3}}$), 1.86 (qt, J=7.4, 6.50 Hz, 2 H, -O-CH_2-$\underline{\mathbf{CH_2}}$-$CH_3$) 4.02 (t, *J*=6.5 Hz, 2 H, -O-$\underline{\mathbf{CH_2}}$-$CH_2$-), 6.99 (d, *J*=9.0 Hz, 2 H, Ar), 7.40 (d, *J*=9.4 Hz, 2 H, Ar), 8.13 (d, *J*=9.0 Hz, 2 H, Ar), 8.29 (d, *J*=8.9 Hz, 2 H, Ar).
^{13}C NMR: (75 MHz, Chloroform-d) δ ppm: 10.42, 22.42, 69.90, 114.54, 120.44, 122.63, 125.15, 132.49, 145.24, 156.00, 163.91, 164.08.

***NxBeO4** – 4-Nitrophenyl-4-butoxybenzoate:*
Yield: 8.34 g, 80.4 %.
Melting point: 61.2 – 62.6 °C.
Infrared $\bar{\nu}$ cm^{-1}: 3086 (sp^2 C-H); 2969, 2944, 2877 (sp^3 C-H); 1734 (C=O); 1606, 1593, 1490 (Ar C=C); 1527 (NO_2); 842, 827 (p. disubs. benzene C-H).
^{1}H NMR: (300 MHz, Chloroform-d) δ ppm: 1.02 (t, *J*=7.4 Hz, 3 H, -CH_2-**CH_3**), 1.54 (qt, *J*=7.5, 7.2 Hz, 2 H, -CH_2-**CH_2**-CH_3), 1.84 (tt, *J*=7.5, 6.5 Hz, 2 H, -O-CH_2-**CH_2**-CH_2-), 4.08 (t, *J*=6.5 Hz, 2 H, -O-**CH_2**-CH_2-), 7.01 (d, *J*=9.0 Hz, 2 H, Ar), 7.42 (d, *J*=9.2 Hz, 2 H, Ar), 8.15 (d, *J*=9.0 Hz, 2 H, Ar), 8.33 (d, *J*=9.2 Hz, 2 H, Ar).
^{13}C NMR: (75 MHz, Chloroform-d) δ ppm: 13.84, 19.20, 31.10, 68.12, 114.51, 120.37, 122.70, 125.23, 132.53, 145.22, 155.97, 163.98, 164.07.

***NxBeO5** – 4-Nitrophenyl-4-pentoxybenzoate*:
Yield: 7.65 g, 70.0 %.
Melting point: 61.9 – 62.4 °C.
Infrared $\bar{\nu}$ cm^{-1}: 3085 (sp^2 C-H); 2963, 2944, 2875, 2860 (sp^3 C-H); 1733 (C=O); 1607, 1588, 1489 (Ar C=C); 1513 (NO_2); 846 (p. disubs. benzene C-H).
^{1}H NMR: (300 MHz, Chloroform-d) δ ppm: 0.97 (t, *J*=7.2 Hz, 3 H, -CH_2-**CH_3**), 1.33 – 1.56 (m, 4 H, -CH_2-**CH_2**-**CH_2**-CH_3), 1.85 (tt, *J*=7.3, 6.6 Hz, 2 H, -O-CH_2-**CH_2**-CH_2-), 4.07 (t, *J*=6.6 Hz, 2 H, -O-**CH_2**-CH_2-), 7.00 (d, *J*=9.0 Hz, 2 H, Ar), 7.41 (d, J=9.2 Hz, 2 H, Ar), 8.14 (d, *J*=9.0 Hz, 2 H, Ar), 8.31 (d, J=9.2 Hz, 2 H).
^{13}C NMR: (75 MHz, Chloroform-d) δ ppm: 14.02, 22.45 28.13, 28.77, 68.43, 114.51, 120.38, 122.67, 125.19, 132.51, 145.21, 155.98, 163.93, 164.07.
***NxBeO6** – 4-Nitrophenyl-4-hexyloxybenzoate*:
Yield: 2.02 g, 80.1 %.
Melting point: 64.7 – 65.5 °C.
Infrared $\bar{\nu}$ cm^{-1}: 3075 (sp^2 C-H); 2930, 2866 (sp^3 C-H); 1738 (C=O); 1606, 1591, 1489 (Ar C=C); 1513 (NO_2); 850 (p. disubs. benzene C-H).
^{1}H NMR: (300 MHz, Chloroform-d) δ ppm: 0.93 (t, *J*=7.0 Hz, 3 H, -CH_2-**CH_3**), 1.28 – 1.58 (m, 6 H, -CH_2-**CH_2**-**CH_2**-**CH_2**-CH_3), 1.84 (tt, *J*=7.4, 6.6 Hz, 2 H, -O-CH_2-**CH_2**-CH_2-), 4.06 (t, *J*=6.6 Hz, 2 H, -O-**CH_2**-CH_2-), 7.00 (d, *J*=8.9 Hz, 2 H, Ar), 7.41 (d, J=9.2 Hz, 2 H, Ar), 8.14 (d, *J*=8.9 Hz, 2 H, Ar), 8.31 (d, J=9.2 Hz, 2 H).
^{13}C NMR: (75 MHz, Chloroform-d) δ ppm: 14.07, 22.61, 25.67, 29.04, 31.56, 68.44, 114.51, 120.35, 122.69, 125.20, 132.51, 145.18, 155.98, 163.95, 164.07.

***NxBeO7** – 4-Nitrophenyl-4-heptyloxybenzoate*:
Yield: 9.34 g, 79.4 %.
Melting point: 57.9 °C (Cr-N), 60.9 °C (N-I).
Infrared $\bar{\nu}$ cm^{-1}: 3080 (sp^2 C-H); 2925, 2860 (sp^3 C-H); 1732 (C=O); 1608, 1590, 1490 (Ar C=C); 1519 (NO_2); 848, 837 (p. disubs. benzene C-H).
^{1}H NMR: (300 MHz, Chloroform-d) δ ppm: 0.92 (t, *J*=6.8 Hz, 3 H, -CH_2-**CH_3**), 1.25 – 1.57 (m, 8 H, -CH_2-**CH_2**-**CH_2**-**CH_2**-**CH_2**-CH_3), 1.85 (tt, *J*=7.3, 6.6 Hz, 2 H, -O-CH_2-**CH_2**-CH_2-), 4.07 (t, *J*=6.5 Hz, 2 H, -O-**CH_2**-CH_2-), 7.01 (d, *J*=8.9 Hz, 2 H, Ar), 7.42 (d, *J*=9.2 Hz, 2 H, Ar), 8.15 (d, *J*=9.0 Hz, 2 H, Ar), 8.33 (d, *J*=9.2 Hz, 2 H, Ar).
^{13}C NMR: (75 MHz, Chloroform-d) δ ppm: 14.12, 22.63, 25.95, 29.05, 29.08, 31.78, 68.45, 114.51, 120.35, 122.70, 125.23, 132.53, 145.21, 155.98, 163.98, 164.07.

***NxBeO8** – 4-Nitrophenyl-4-octyloxybenzoate:*
Yield: 5.43 g, 73.5 %.
Melting point: 50.5 °C (Cr-SmA), 60.6 °C (SmA-N), 67.3 °C (N-I).
Infrared $\bar{\nu}$ cm^{-1}: 3085 (sp^2 C-H); 2927, 2871 (sp^3 C-H); 1736 (C=O); 1605, 1591, 1488 (Ar C=C); 1510 (NO_2); 843 (p. disubs. benzene C-H).
^{1}H NMR: (300 MHz, Chloroform-d) δ ppm: 0.92 (t, *J*=7.2 Hz, 3 H, -CH$_2$-**CH$_3$**), 1.23 – 1.58 (m, 10 H, -CH$_2$-**CH$_2$**-**CH$_2$**-**CH$_2$**-**CH$_2$**-**CH$_2$**-CH$_3$), 1.85 (tt, *J*=7.0, 6.6 Hz, 2 H, -O-CH$_2$-**CH$_2$**-CH$_2$-), 4.08 (t, *J*=6.6 Hz, 2 H, -O-**CH$_2$**-CH$_2$-), 7.01 (d, *J*=9.0 Hz, 2 H, Ar), 7.42 (d, *J*=9.0 Hz, 2 H, Ar), 8.15 (d, *J*=8.7 Hz, 2 H, Ar), 8.33 (d, *J*=9.4 Hz, 2 H, Ar).
^{13}C NMR: (75 MHz, Chloroform-d) δ ppm: 14.05, 22.63, 25.97, 29.06, 29.19, 29.30, 31.78, 68.46, 114.54, 120.45, 122.63, 125.18, 132.49, 145.28, 155.99, 163.93, 164.09.

***NxBeO9** – 4-Nitrophenyl-4-nonyloxybenzoate:*
Yield: 5.10 g, 82.2 %.
Melting point: 53.8 °C (Cr-SmA), 67.9 °C (SmA-I).
Infrared $\bar{\nu}$ cm^{-1}: 3087 (sp^2 C-H); 2964, 2953, 2943, 2919, 2853 (sp^3 C-H); 1733 (C=O); 1607, 1594, 1490 (Ar C=C); 1511 (NO_2); 842, 831 (p. disubs. benzene C-H).
^{1}H NMR: (300 MHz, Chloroform-d) δ ppm: 0.92 (t, *J*=7.2 Hz, 3 H, -CH$_2$-**CH$_3$**), 1.31 – 1.58 (m, 12 H, -CH$_2$-**CH$_2$**-**CH$_2$**-**CH$_2$**-**CH$_2$**-**CH$_2$**-**CH$_2$**-CH$_3$), 1.86 (tt, *J*=7.2, 6.5 Hz, 2 H, -O-CH$_2$-**CH$_2$**-CH$_2$-), 4.08 (t, *J*=6.5 Hz, 2 H, -O-**CH$_2$**-CH$_2$-), 7.02 (d, *J*=8.9 Hz, 2 H, Ar), 7.43 (d, *J*=8.7 Hz, 2 H, Ar), 8.15 (d, *J*=9.0 Hz, 2 H, Ar), 8.31 (d, *J*=9.2 Hz, 2 H, Ar).
^{13}C NMR: (75 MHz, Chloroform-d) δ ppm: 13.99, 22.60, 25.91, 29.02, 29.18, 29.30, 29.45, 31.81, 68.43, 114.51, 120.35, 122.59, 125.09, 132.44, 145.18, 155.96, 163.93, 164.06.

***NxBeO10** – 4-Nitrophenyl 4-decyloxybenzoate:*
Yield: 5.94 g, 85.0 %.
Melting point: 58.9 °C (Cr-SmA), 76.4 (SmA-I) °C.
Infrared $\bar{\nu}$ cm^{-1}: 3086 (sp^2 C-H); 2954, 2942, 2918, 2873, 2852 (sp^3 C-H); 1732 (C=O); 1607, 1594, 1491 (Ar C=C); 1511 (NO_2); 841 (p. disubs. benzene C-H).
^{1}H NMR: (300 MHz, Chloroform-d) δ ppm: 0.90 (t, *J*=6.8 Hz, 3 H, -CH$_2$-**CH$_3$**), 1.20 – 1.57 (m, 14 H, -CH$_2$-**CH$_2$**-**CH$_2$**-**CH$_2$**-**CH$_2$**-**CH$_2$**-**CH$_2$**-**CH$_2$**-CH$_3$), 1.85 (tt, *J*=7.5, 6.5 Hz, 2 H, -O-CH$_2$-**CH$_2$**-CH$_2$-), 4.07 (t, *J*=6.5 Hz, 2 H, -O-**CH$_2$**-CH$_2$-), 7.00 (d, *J*=8.9 Hz, 2 H, Ar), 7.42 (d, *J*=9.2 Hz, 2 H, Ar), 8.15 (d, *J*=8.9 Hz, 2 H, Ar), 8.33 (d, *J*=9.0 Hz, 2 H, Ar).
^{13}C NMR: (75 MHz, Chloroform-d) δ ppm: 14.15, 22.71, 25.99, 29.08, 29.35, 29.38, 29.57, 31.91, 68.45, 114.51, 120.36, 122.68, 125.21, 132.51, 145.21, 155.98, 163.95, 164.07.

Synthesis of 4-aminophenyl-4’-(alkoxy)benzoate esters (H2NBeOn)

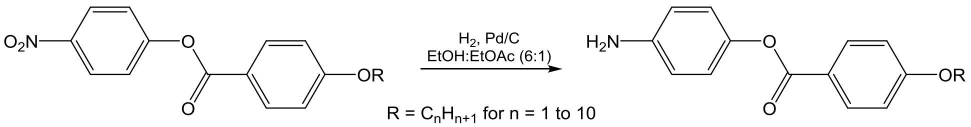

Supplementary Figure 14: Synthesis of 4-aminophenyl-4’-(alkoxy)benzoate esters.

Selective reduction of 4-nitrophenyl-4’-(alkoxy)benzoate esters over a palladium/activated charcoal catalyst gave the 4-aminophenyl-4’-(alkoxy)benzoate intermediates, as outlined in Supplementary Figure 14. In a flask, the required 4-nitrophenyl-4’-(alkoxy)benzoate (1 mol. eq.) was dissolved in ethyl acetate (50 ml) and ethanol (300 ml), with 10 % Pd/C, 53% wet (0.01 mol. eq. of Pd), and flushed with argon before evacuating under vacuum. The actual quantities used for each reduction are given in Table S2. Following de-gassing, hydrogen gas was bubbled into the vessel *via* a balloon at ambient pressure and temperature. After 12 h, solid Pd/C was filtered out through a Celite plug and washed with ethyl acetate (300 ml), and the solvent removed *via* rotary evaporation to give the desired 4-aminophenyl-4-(alkoxy)benzoate ester. The reported yields of the beige crystals are given below after recrystallisation from boiling ethanol (30 ml/g of ester), and drying overnight *in vacuo* at 50 °C.

Supplementary Table 2. Quantities of reagents used in the synthesis of 4-amino phenyl-4-(alkyloxy) benzoate intermediates.

	**4-Nitrophenyl 4’-(alkyloxy)benzoate used:**	**Quantity of 4-nitrophenyl 4’-(alkyloxy)benzoate used:**	**Quantity of 10% Pd/C, 53 % wet used:**
n = 1	4-Nitrophenyl 4’-methoxybenzoate	7.018 g, 2.57 x 10^{-2} mol, 1.0 eq.	705 mg, 3.11 x 10^{-4} mol, 0.012 eq.
n = 2	4-Nitrophenyl 4’-ethoxybenzoate	8.014 g, 2.79 x 10^{-2} mol, 1.0 eq.	774 mg, 3.42 x 10^{-4} mol, 0.012 eq.
n = 3	4-Nitrophenyl 4’-propoxybenzoate	4.626 g, 1.54 x 10^{-2} mol, 1.0 eq.	441 mg, 1.95 x 10^{-4} mol, 0.013 eq.
n = 4	4-Nitrophenyl 4’-butoxybenzoate	7.853 g, 2.49 x 10^{-2} mol, 1.0 eq.	701 mg, 3.10 x 10^{-4} mol, 0.012 eq.
n = 5	4-Nitrophenyl 4’-pentoxybenzoate	7.012 g, 2.13 x 10^{-2} mol, 1.0 eq.	584 mg, 2.58 x 10^{-4} mol, 0.012 eq.
n = 6	4-Nitrophenyl 4’-hexyloxybenzoate	1.722 g, 5.01 x 10^{-3} mol, 1.0 eq.	142 mg, 6.27 x 10^{-5} mol, 0.013 eq.
n = 7	4-Nitrophenyl 4’-heptyloxybenzoate	8.721 g, 2.44 x 10^{-2} mol, 1.0 eq.	681 mg, 3.11 x 10^{-4} mol, 0.012 eq.
n = 8	4-Nitrophenyl 4’-octyloxybenzoate	4.978 g, 1.34 x 10^{-2} mol, 1.0 eq.	378 mg, 1.67 x 10^{-4} mol, 0.012 eq.
n = 9	4-Nitrophenyl 4’-nonyloxybenzoate	4.632 g, 1.20 x 10^{-2} mol, 1.0 eq.	338 mg, 1.49 x 10^{-4} mol, 0.012 eq.
n = 10	4-Nitrophenyl 4’-decyloxybenzoate	5.573 g, 1.39 x 10^{-2} mol, 1.0 eq.	392 mg, 1.73 x 10^{-4} mol, 0.012 eq.

***H_2NBeO1** – 4-Aminophenyl-4’-methoxybenzoate:*

Yield: 6.14 g, 98.3 %.
Melting point: 140.9 °C.
Infrared $\bar{\nu}$ cm^{-1}: 3464, 3379 (N-H); 3019 (sp^2 C-H); 2977, 2922, 2843 (sp^3 C-H); 1713 (C=O); 1606, 1578, 1509 (Ar C=C); 1264 (Ar C-O); 843, 821 (p. disubs. benzene C-H).
^{1}H NMR: (300 MHz, Chloroform-d) δ ppm: 3.70 (br. s., 2 H, **H_2N**-Ar-), 3.90 (s, 3H, -O-**CH_3**), 6.71 (d, *J*=8.7 Hz, 2 H, Ar), 6.91 - 7.11 (m, 4 H, Ar), 8.17 (d, *J*=8.8 Hz, 2 H, Ar).
^{13}C NMR: (75 MHz, Chloroform-d) δ ppm: 55.52, 113.81, 115.68, 122.16, 122.35, 132.20, 143.10, 144.31, 163.77, 165.51.

***H_2NBeO2** – 4-Aminophenyl-4'-ethoxybenzoate:*

Yield: 7.01 g, 97.7 %.
Melting point: 152.2 °C.
Infrared $\bar{\nu}$ cm^{-1}: 3493, 3395 (N-H); 2978, 2933 (sp^3 C-H); 1713 (C=O); 1605, 1511 (Ar C=C); 1257 (Ar C-O); 848, 819 (p. disubs. benzene C-H).
^{1}H NMR: (300 MHz, Chloroform-d) δ ppm: 1.48 (t, J=7.0 Hz, 3 H, -O-CH_2-**CH_3**), 3.80 (br. s., 2 H, **H_2N**-Ar-), 4.13 (q, J=7.0 Hz, 2 H, -O-**CH_2**-CH_3), 6.72 (d, *J*=8.7 Hz, 2 H, Ar), 6.92 - 7.07 (m, 4 H, Ar), 8.15 (d, *J*=8.9 Hz, 2 H, Ar).
^{13}C NMR: (75 MHz, Chloroform-d) δ ppm: 14.71, 63.79, 114.19, 115.69, 121.90, 122.38, 132.20, 143.16, 144.17, 163.16, 165.53.

***H_2NBeO3** – 4-Aminophenyl-4'-propoxybenzoate:*

Yield: 4.01 g, 96.2 %.
Melting point: 133.4 °C.
Infrared $\bar{\nu}$ cm^{-1}: 3476, 3388 (N-H); 2966, 2912, 2877 (sp^3 C-H); 1719 (C=O); 1604, 1510, 1472 (Ar C=C); 1262 (Ar C-O); 847, 826 (p. disubs. benzene C-H).
^{1}H NMR: (300 MHz, Chloroform-d) δ ppm: 1.07 (t, J=7.4 Hz, 3 H, -CH_2-**CH_3**), 1.86 (qt, J=7.3, 6.5 Hz, 2 H, -O-CH_2-**CH_2**-CH_3), 3.58 (br. s., 2 H, **H_2N**-Ar-), 3.99 (t, J=6.5 Hz, 2 H, -O-**CH_2**-CH_2-), 6.69 (d, *J*=8.7 Hz, 2 H, Ar), 6.89 - 7.06 (m, 4 H, Ar), 8.15 (d, *J*=8.9 Hz, 2 H, Ar).
^{13}C NMR: (75 MHz, Chloroform-d) δ ppm: 10.50, 22.47, 69.79, 114.27, 115.70, 121.83, 122.34, 132.19, 143.07, 144.36, 163.30, 165.62.

***H_2NBeO4** – 4-Aminophenyl-4'-butoxybenzoate:*

Yield: 7.00 g, 98.5 %.
Melting point: 110.0 °C.
Infrared $\bar{\nu}$ cm^{-1}: 3453, 3365 (N-H); 2953, 2939, 2875 (sp^3 C-H); 1708 (C=O); 1604, 1578, 1507, 1476 (Ar C=C); 1252 (Ar C-O); 852, 826 (p. disubs. benzene C-H).
^{1}H NMR: (300 MHz, Chloroform-d) δ ppm: 1.02 (t, *J*=7.4 Hz, 3 H, -CH_2-**CH_3**), 1.54 (tq, *J*=7.4, 7.3 Hz, 2 H, -CH_2-**CH_2**-CH_3-), 1.83 (tt, *J*=7.4, 6.4 Hz, 2 H, -O-CH_2-**CH_2**-CH_2-), 3.69 (br. s., 2 H, **H_2N**-Ar-), 4.06 (t, *J*=6.4 Hz, 2 H, -O-**CH_2**-CH_2-), 6.71 (d, *J*=8.7 Hz, 2 H, Ar), 6.92 - 7.07 (m, 4 H, Ar), 8.15 (d, *J*=8.7 Hz, 2 H, Ar).
^{13}C NMR: (75 MHz, Chloroform-d) δ ppm: 13.87, 19.22, 31.16, 67.98, 114.24, 115.69, 121.85, 122.37, 132.18, 143.16, 144.22, 163.39, 165.56.

***H_2NBeO5** – 4-Aminophenyl-4'-pentoxybenzoate:*

Yield: 6.16 g, 96.6 %.
Melting point: 94.0 °C.
Infrared $\bar{\nu}$ cm^{-1}: 3466, 3378 (N-H); 2948, 2867 (sp^3 C-H); 1718 (C=O); 1603, 1579, 1511 (Ar C=C); 1256 (Ar C-O); 823 (p. disubs. benzene C-H).
^{1}H NMR: (300 MHz, Chloroform-d) δ ppm: 0.97 (t, *J*=7.0 Hz, 3 H, -CH_2-**CH_3**), 1.34 – 1.57 (m, 4 H, -CH_2-**CH_2**-**CH_2**-CH_3), 1.85 (tt, *J*=7.3, 6.5 Hz, 2 H, -O-CH_2-**CH_2**-CH_2-), 3.69 (br. s., 2 H, **H_2N**-Ar-), 4.05 (t, *J*=6.5 Hz, 2 H, -O-**CH_2**-CH_2-), 6.72 (d, *J*=8.7 Hz, 2 H, Ar), 6.91 - 7.07 (m, 4 H, Ar), 8.13 (d, *J*=8.9 Hz, 2 H, Ar).
^{13}C NMR: (75 MHz, Chloroform-d) δ ppm: 14.07, 22.47, 28.15, 28.82, 68.28, 114.22, 115.70, 121.82, 122.38, 132.19, 143.16, 144.17, 163.37, 165.56.

***H$_2$NBeO6** – 4-Aminophenyl-4'-hexyloxybenzoate:*

Yield: 1.49 g, 94.5 %.

Melting point: 78.5 °C.

Infrared $\bar{\nu}$ cm^{-1}: 3461, 3374 (N-H); 2955, 2929, 2869 (sp^3 C-H); 1713 (C=O); 1605, 1581, 1512 (Ar C=C); 1253 (Ar C-O); 843, 826 (p. disubs. benzene C-H).

^{1}H NMR: (300 MHz, Chloroform-d) δ ppm: 0.93 (t, *J*=7.0 Hz, 3 H, -CH$_2$-**CH$_3$**), 1.31 – 1.57 (m, 6 H, -CH$_2$-**CH$_2$**-**CH$_2$**-**CH$_2$**-CH$_3$), 1.83 (tt, *J*=7.2, 6.5 Hz, 2 H, -O-CH$_2$-**CH$_2$**-CH$_2$-), 3.75 (br. s., 2 H, **H$_2$N**-Ar-), 4.04 (t, *J*=6.5 Hz, 2 H, -O-**CH$_2$**-CH$_2$-), 6.70 (d, *J*=8.9 Hz, 2 H, Ar), 6.91 - 7.04 (m, 4 H, Ar), 8.13 (d, *J*=8.9 Hz, 2 H, Ar).

^{13}C NMR: (75 MHz, Chloroform-d) δ ppm: 14.03, 22.59, 25.67, 29.08, 31.55, 68.30, 114.23, 115.68, 121.87, 122.35, 132.17, 143.21, 144.15, 163.38, 165.51.

***H$_2$NBeO7** – 4-Aminophenyl-4'-heptyloxybenzoate:*

Yield: 7.77 g, 97.3 %.

Melting point: 85.7 °C.

Infrared $\bar{\nu}$ cm^{-1}: 3463, 3375 (N-H); 2949, 2926, 2868 (sp^3 C-H); 1714 (C=O); 1605, 1581, 1511 (Ar C=C); 1254 (Ar C-O); 850, 826 (p. disubs. benzene C-H).

^{1}H NMR: (300 MHz, Chloroform-d) δ ppm: 0.93 (t, *J*=6.6 Hz, 3 H, -CH$_2$-**CH$_3$**), 1.30 – 1.53 (m, 8 H, -CH$_2$-**CH$_2$**-**CH$_2$**-**CH$_2$**-**CH$_2$**-CH$_3$), 1.84 (tt, *J*=7.5, 6.5 Hz, 2 H, -O-CH$_2$-**CH$_2$**-CH$_2$-), 3.76 (br. s, 2 H, **H$_2$N**-Ar-), 4.05 (t, *J*=6.5 Hz, 2 H, -O-**CH$_2$**-CH$_2$-), 6.72 (d, *J*=8.9 Hz, 2 H, Ar), 6.94 – 7.04 (m, 4 H, Ar), 8.15 (d, *J*=8.9 Hz, 2 H, Ar).

^{13}C NMR: (75 MHz, Chloroform-d) δ ppm: 14.15, 22.64, 25.97, 29.07, 29.13, 31.80, 68.30, 114.22, 115.69, 121.82, 122.38, 132.19, 143.15, 144.20, 163.38, 165.56.

***H$_2$NBeO8** – 4-Aminophenyl-4'-octyloxybenzoate:*

Yield: 4.37 g, 95.5 %.

Melting point: 93.3 °C.

Infrared $\bar{\nu}$ cm^{-1}: 3468, 3374 (N-H); 2958, 2934, 2916, 2850 (sp^3 C-H); 1711 (C=O); 1604, 1577, 1510 (Ar C=C); 1254 (Ar C-O); 844, 823 (p. disubs. benzene C-H).

^{1}H NMR: (300 MHz, Chloroform-d) δ ppm: 0.92 (t, *J*=7.0 Hz, 3 H, -CH$_2$-**CH$_3$**), 1.22 – 1.60 (m, 10 H, -CH$_2$-**CH$_2$**-**CH$_2$**-**CH$_2$**-**CH$_2$**-**CH$_2$**-CH$_3$), 1.84 (tt, *J*=7.0, 6.6 Hz, 2 H, -O-CH$_2$-**CH$_2$**-CH$_2$-), 3.69 (br. s, 2 H, **H$_2$N**-Ar-), 4.05 (t, *J*=6.6 Hz, 2 H, -O-**CH$_2$**-CH$_2$-), 6.72 (d, *J*=8.9 Hz, 2 H, Ar), 6.92 – 7.06 (m, 4 H, Ar), 8.15 (d, *J*=8.9 Hz, 2 H, Ar).

^{13}C NMR: (75 MHz, Chloroform-d) δ ppm: 14.15, 22.69, 26.02, 29.13, 29.26, 29.36, 31.83, 68.30, 114.22, 115.69, 121.82, 122.38, 132.19, 143.16, 144.19, 163.38, 165.56.

***H$_2$NBeO9** – 4-Aminophenyl-4'-nonyloxybenzoate:*

Yield: 3.99 g, 93.5 %.

Melting point: 90.3 °C.

Infrared $\bar{\nu}$ cm^{-1}: 3462, 3374 (N-H); 2947, 2918, 2868, 2849 (sp^3 C-H); 1715 (C=O); 1605, 1580, 1511 (Ar C=C); 1257 (Ar C-O); 848, 824 (p. disubs. benzene C-H).

^{1}H NMR: (300 MHz, Chloroform-d) δ ppm: 0.92 (t, *J*=7.2 Hz, 3 H, -CH$_2$-**CH$_3$**), 1.26 – 1.51 (m, 12 H, -CH$_2$-**CH$_2$**-**CH$_2$**-**CH$_2$**-**CH$_2$**-**CH$_2$**-**CH$_2$**-CH$_3$), 1.84 (tt, *J*=7.2, 6.6 Hz, 2 H, -O-CH$_2$-**CH$_2$**-CH$_2$-), 3.74 (br. s, 2 H, **H$_2$N**-Ar-), 4.05 (t, *J*=6.6 Hz, 2 H, -O-**CH$_2$**-CH$_2$-), 6.72 (d, *J*=8.5 Hz, 2 H, Ar), 6.90 – 7.10 (m, 4 H, Ar), 8.15 (d, *J*=9.0 Hz, 2 H, Ar).

^{13}C NMR: (75 MHz, Chloroform-d) δ ppm: 14.17, 22.71, 26.00, 29.12, 29.29, 29.40, 29.55, 31.90, 68.30, 114.22, 115.70, 121.80, 122.38, 132.19, 143.16, 144.17, 163.37, 165.57.

***H_2NBeO10** – 4-Aminophenyl-4'-decyloxybenzoate:*
Yield: 4.78 g, 92.8 %.
Melting point: 91.5 °C.
Infrared $\bar{\nu}$ cm^{-1}: 3457, 3371 (N-H); 2953, 2919, 2850 (sp^3 C-H); 1712 (C=O); 1605, 1579, 1511 (Ar C=C); 1256 (Ar C-O); 846, 826 (p. disubs. benzene C-H)
^1H NMR: (300 MHz, Chloroform-d) δ ppm: 0.93 (t, *J*=7.0 Hz, 3 H, -CH_2-**CH_3**), 1.27 – 1.62 (m, 14 H, -CH_2-**CH_2**-**CH_2**-**CH_2**-**CH_2**-**CH_2**-**CH_2**-**CH_2**-CH_3), 1.85 (tt, *J*=7.0, 6.5 Hz, 2 H, -O-CH_2-**CH_2**-CH_2-), 3.70 (br. s, 2 H, **H_2N**-Ar-), 4.05 (t, *J*=6.5 Hz, 2 H, -O-**CH_2**-CH_2-), 6.71 (d, *J*=9.0 Hz, 2 H, Ar), 6.93 – 7.07 (m, 4 H, Ar), 8.16 (d, *J*=8.9 Hz, 2 H, Ar).
^{13}C NMR: (75 MHz, Chloroform-d) δ ppm: 14.20, 22.74, 23.01, 26.03, 29.14, 29.38, 29.42, 29.61, 31.95, 68.30, 114.24, 115.68, 121.84, 122.37, 132.19, 143.12, 144.28, 163.39, 165.56.

Synthesis of 4-[{[4-({6-[4-(4-cyanophenyl)phenyl]hexyl}oxy)phenyl]-methylidene} amino]phenyl 4-alkoxybenzoates (CB6OIBeO*n*).

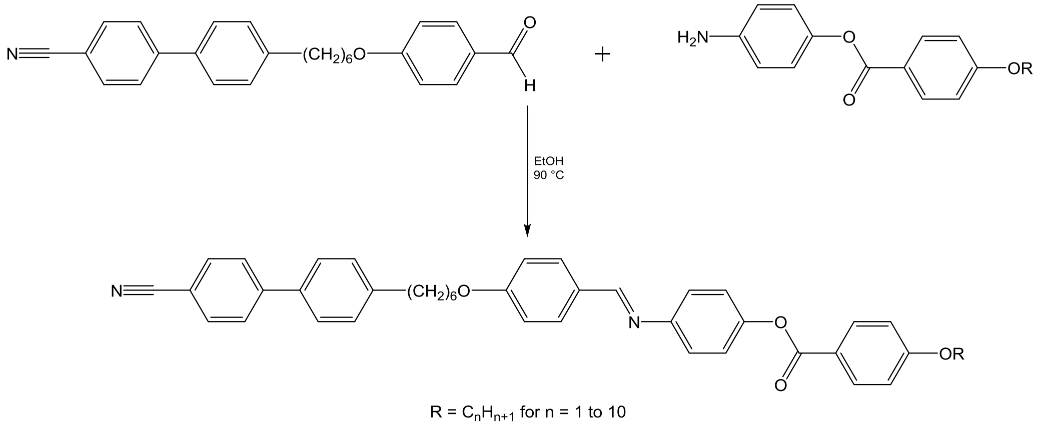

Supplementary Figure 15: Synthesis of 4-[{[4-({6-[4-(4-cyanophenyl)phenyl]hexyl} oxy)phenyl] methylidene}amino]phenyl 4-alkoxybenzoates.

The synthesis of 4-[{[4-({6-[4-(4-cyanophenyl)phenyl]hexyl}oxy)phenyl]methylidene} amino]phenyl 4-alkoxybenzoate dimers followed a general Schiff base synthesis, as outlined in Supplementary Figure 15.
4-{4-[6-(4-formylphenoxy)hexyl]phenyl}benzonitrile (1 mol. eq.) and the desired 4-aminophenyl-4-(alkoxy)benzoate ester (1 mol. eq.) were combined in ethanol (10 ml) and refluxed with stirring for 2 h. Exact quantities of reagents used are given in Table S3. The pale yellow solutions were left to cool to room temperature, and then to ~0 °C for 30 min. The pale yellow crystals were collected *via* vacuum filtration and recrystallised from boiling ethyl acetate (10 ml) with petroleum ether 40-60 as a co-solvent (5 to 10 drops), with cooling overnight to -20 °C. Recrystallisation was repeated three times, and the compounds dried *in vacuo* overnight at 50 °C to give the dimers in the reported yields. It

was not possible to obtain ^{13}C NMR spectra for the complete series because the compounds degraded during the measurement.

Supplementary Table 3. Quantities of reagents used in the synthesis of 4-[{[4-({6-[4-(4-cyanophenyl) phenyl]hexyl}oxy)phenyl]methylidene}amino]phenyl 4-alkoxybenzoate dimers

	**4-Aminophenyl 4-(alkyloxy)benzoate used:**	**Quantity of 4-Aminophenyl 4-(alkoxy)benzoate used:**	**Quantity of CB6OIPhK used:**
**n = 1**	4-Aminophenyl 4-methoxybenzoate	192 mg, 7.89 x 10^{-4} mol, 1.0 eq.	302 mg, 7.88 x 10^{-4} mol, 1.0 eq.
**n = 2**	4-Aminophenyl 4-ethoxybenzoate	202 mg, 7.85 x 10^{-4} mol, 1.0 eq.	298 mg, 7.77 x 10^{-4} mol, 1.0 eq.
**n = 3**	4-Aminophenyl 4-propoxybenzoate	210 mg, 7.74 x 10^{-4} mol, 1.0 eq.	301 mg, 7.85 x 10^{-4} mol, 1.0 eq.
**n = 4**	4-Aminophenyl 4-butoxybenzoate	226 mg, 7.92 x 10^{-4} mol, 1.0 eq.	300 mg, 7.82 x 10^{-4} mol, 1.0 eq.
**n = 5**	4-Aminophenyl 4-pentoxybenzoate	232 mg, 7.75 x 10^{-4} mol, 1.0 eq.	304 mg, 7.93 x 10^{-4} mol, 1.0 eq.
**n = 6**	4-Aminophenyl 4-hexyloxybenzoate	247 mg, 7.88 x 10^{-4} mol, 1.0 eq.	297 mg, 7.74 x 10^{-4} mol, 1.0 eq.
**n = 7**	4-Aminophenyl-4-heptyloxybenzoate	256 mg, 7.82 x 10^{-4} mol, 1.0 eq.	295 mg, 7.69 x 10^{-4} mol, 1.0 eq.
**n = 8**	4-Aminophenyl-4-octyloxybenzoate	264 mg, 7.73 x 10^{-4} mol, 1.0 eq.	301 mg, 7.85 x 10^{-4} mol, 1.0 eq.
**n = 9**	4-Aminophenyl-4-nonyloxybenzoate	279 mg, 7.85 x 10^{-4} mol, 1.0 eq.	303 mg, 7.90 x 10^{-4} mol, 1.0 eq.
**n = 10**	4-Aminophenyl-4-decyloxybenzoate	285 mg, 7.71 x 10^{-4} mol, 1.0 eq.	302 mg, 7.88 x 10^{-4} mol, 1.0 eq.

CB6OIBeO1 *- 4-[{[4-({6-[4-(4-Cyanophenyl)phenyl]hexyl}oxy)phenyl]methylidene}amino] phenyl 4-methoxybenzoate:*

Yield: 287 mg, 59.9 %.

Infrared $\bar{\nu}$ cm^{-1}: 3022 (sp^2 C-H); 2929, 2853 (sp^3 C-H); 2221 (C≡N); 1723 (C=O); 1603, 1572, 1511, 1495 (Ar C=C); 1252 (Ar C-O); 841, 828 (p. disubs. benzene C-H).

^1H NMR (300 MHz, DMSO-d_6) δ ppm: 1.30 - 1.54 (m, 4 H, -CH_2-CH_2-**CH_2**-**CH_2**-CH_2-CH_2-O-), 1.63 (quin, *J*=7.5, 2 H, -CH_2-**CH_2**-CH_2-CH_2-CH_2-CH_2-O-), 1.74 (tt, *J*=7.2, 6.4 Hz, 2 H, -CH_2-CH_2-CH_2-CH_2-**CH_2**-CH_2-O-), 2.64 (t, *J*=7.5 Hz, 2 H, -Ar-**CH_2**-CH_2-), 3.88 (s, 3 H, -Ar-O-**CH_3**), 4.04 (t, *J*=6.4 Hz, 2 H, -CH_2-**CH_2**-O-), 7.05 (d, *J*=8.7 Hz, 2 H, Ar), 7.14 (d, *J*=9.0 Hz, 2 H, Ar), 7.26 - 7.36 (m, 6 H, Ar), 7.66 (d, *J*=8.3 Hz, 2 H, Ar), 7.80 - 7.93 (m, 6 H, Ar), 8.10 (d, *J*=8.9 Hz, 2 H, Ar), 8.57 (s, 1 H, -**CH**=N-).

^{13}C NMR: (75 MHz, DMSO-d_6) δ ppm: 25.23, 28.28, 28.45, 30.67, 34.64, 55.62, 67.65, 109.63, 114.25, 114.68, 118.89, 120.99, 121.80, 122.56, 126.90, 127.23, 128.71, 129.10, 130.47, 131.96, 132.76, 135.56, 143.14, 144.54, 148.43, 149.30, 160.03, 161.36, 163.70, 164.28.

Elemental analysis: Calculated for $C_{40}H_{36}N_2O_4$: C, 78.92 %, H, 5.96 %, N, 4.60 %. Found: C, 79.01 %, H, 6.00 %, N, 4.47 %.

***CB6OIBeO2 - 4-[{[4-({6-[4-(4-Cyanophenyl)phenyl]hexyl}oxy)phenyl]methylidene}amino] phenyl 4-ethoxybenzoate*:**

Yield: 264 mg, 54.6 %.

Infrared $\bar{\nu}$ cm^{-1}: 3062 (sp^2 C-H); 2986, 2975, 2940, 2855 (sp^3 C-H); 2230 (C≡N); 1721 (C=O); 1603, 1571, 1512, 1495 (Ar C=C); 1256 (Ar C-O); 852, 837 (p. disubs. benzene C-H).

^{1}H NMR (300 MHz, DMSO-d_6) δ ppm: 1.31 - 1.55 (m, 7 H, -CH_2-CH_2-**$\underline{CH_2}$**-**$\underline{CH_2}$**-CH_2-CH_2-O-, -Ar-O-CH_2-**$\underline{CH_3}$**), 1.64 (quin, *J*=7.5, 2 H, -CH_2-**$\underline{CH_2}$**-CH_2-CH_2-CH_2-CH_2-O-), 1.75 (tt, *J*=7.0, 6.4 Hz, 2 H, -CH_2-CH_2-CH_2-CH_2-**$\underline{CH_2}$**-CH_2-O-), 2.65 (t, *J*=7.5 Hz, 2 H, -Ar-**CH_2**-CH_2-), 4.05 (t, *J*=6.4 Hz, 2 H, -CH_2-**$\underline{CH_2}$**-O-), 4.16 (q, J=7.0 Hz, -Ar-O-**$\underline{CH_2}$**-CH_3), 7.06 (d, *J*=8.7 Hz, 2 H, Ar), 7.12 (d, *J*=9.0 Hz, 2 H, Ar), 7.25 - 7.37 (m, 6 H, Ar), 7.66 (d, *J*=8.3 Hz, 2 H, Ar), 7.82 - 7.94 (m, 6 H, Ar), 8.09 (d, *J*=9.0 Hz, 2 H, Ar), 8.57 (s, 1 H, -**$\underline{CH}$**=N-).

Elemental analysis: Calculated for $C_{41}H_{38}N_2O_4$: C, 79.07 %, H, 6.15 %, N, 4.50 %. Found: C, 78.61 %, H, 6.22 %, N, 4.27 %.

***CB6OIBeO3 - 4-[{[4-({6-[4-(4-Cyanophenyl)phenyl]hexyl}oxy)phenyl]methylidene}amino] phenyl 4-propoxybenzoate*:**

Yield: 283 mg, 57.4 %.

Infrared $\bar{\nu}$ cm^{-1}: 3051 (sp^2 C-H); 2969, 2940, 2906, 2867, 2853 (sp^3 C-H); 2223 (C≡N); 1722 (C=O); 1602, 1572, 1509, 1495 (Ar C=C); 1252 (Ar C-O); 845, 811 (p. disubs. benzene C-H).).

^{1}H NMR (300 MHz, DMSO-d_6) δ ppm: 1.00 (t, J=7.4 Hz, 3H, -CH_2-**$\underline{CH_3}$**), 1.32 - 1.56 (m, 4 H, -CH_2-CH_2-**$\underline{CH_2}$**-**$\underline{CH_2}$**-CH_2-CH_2-O-), 1.58 – 1.85 (m, 6 H, -CH_2-**$\underline{CH_2}$**-CH_2-CH_2-**$\underline{CH_2}$**-CH_2-O-, -O-CH_2-**$\underline{CH_2}$**-CH_3), 2.65 (t, *J*=7.4 Hz, 2 H, -Ar-**$\underline{CH_2}$**-CH_2-), 4.02 - 4.09 (two overlapping t, *J*=6.4 Hz, 4 H, -CH_2-CH_2-**$\underline{CH_2}$**-O-, -O-**$\underline{CH_2}$**-CH_2-CH_3), 7.06 (d, *J*=8.7 Hz, 2 H, Ar), 7.13 (d, *J*=9.0 Hz, 2 H, Ar), 7.25 - 7.39 (m, 6 H, Ar), 7.67 (d, *J*=8.3 Hz, 2 H, Ar), 7.82 - 7.95 (m, 6 H, Ar), 8.09 (d, *J*=8.9 Hz, 2 H, Ar), 8.57 (s, 1 H, -**$\underline{CH}$**=N-).

Elemental analysis: Calculated for $C_{42}H_{40}N_2O_4$: C, 79.22 %, H, 6.33 %, N, 4.40 %. Found: C, 78.85 %, H, 6.35 %, N, 4.22 %.

***CB6OIBeO4 - 4-[{[4-({6-[4-(4-Cyanophenyl)phenyl]hexyl}oxy)phenyl]methylidene}amino] phenyl 4-butoxybenzoate*:**

Yield: 301 mg, 59.1 %.

Infrared $\bar{\nu}$ cm^{-1}: 2935, 2868 (sp^3 C-H); 2225 (C≡N); 1723 (C=O); 1603, 1572, 1510, 1494 (Ar C=C); 1249 (Ar C-O); 837 (p. disubs. benzene C-H).

^{1}H NMR (300 MHz, DMSO-d_6) δ ppm: 0.95 (t, *J*=7.4 Hz, 3 H, -CH_2-**$\underline{CH_3}$**), 1.30 - 1.55 (m, 6 H, -CH_2-CH_2-**$\underline{CH_2}$**-**$\underline{CH_2}$**-CH_2-CH_2-O-, -O-CH_2-CH_2-**$\underline{CH_2}$**-CH_3), 1.62 (quin, J=7.4 Hz, 2 H, -Ar-CH_2-**$\underline{CH_2}$**-CH_2-), 1.74 (tt, J=7.3, 6.4 Hz, 4 H, -CH_2-**$\underline{CH_2}$**-CH_2-O-Ar-, -Ar-O-CH_2-**$\underline{CH_2}$**-CH_2-), 2.65 (t, *J*=7.4 Hz, 2 H, -Ar-**$\underline{CH_2}$**-CH_2-), 3.98 - 4.16 (Two overlapping t, J=6.4 Hz, 4 H, -CH_2-**$\underline{CH_2}$**-O-Ar-, -Ar-O-**$\underline{CH_2}$**-CH_2-), 7.05 (d, *J*=8.7 Hz, 2 H, Ar), 7.12 (d, *J*=8.9 Hz, 2 H, Ar), 7.23 - 7.40 (m, 6 H, Ar), 7.66 (d, *J*=8.1 Hz, 2 H, Ar), 7.80 - 7.94 (m, 6 H, Ar), 8.08 (d, *J*=8.9 Hz, 2 H, Ar), 8.57 (s, 1 H, -**$\underline{CH}$**=N-).

Elemental analysis: Calculated for $C_{43}H_{42}N_2O_4$: C, 79.36 %, H, 6.51 %, N, 4.30 %. Found: C, 79.23 %, H, 6.56 %, N, 4.15 %.

CB6OIBeO5 *- 4-[{[4-({6-[4-(4-Cyanophenyl)phenyl]hexyl}oxy)phenyl]methylidene}amino] phenyl 4-pentoxybenzoate*:
Yield: 321 mg, 62.3 %.
Infrared $\bar{\nu}$ cm^{-1}: 2934, 2860 (sp^3 C-H); 2225 (C≡N); 1726 (C=O); 1604, 1573, 1511, 1494 (Ar C=C); 1248 (Ar C-O); 851, 835 (p. disubs. benzene C-H).
^{1}H NMR (300 MHz, DMSO-d_6) δ ppm: 0.91 (t, *J*=7.2 Hz, 3 H, -CH_2-**CH_3**), 1.28 - 1.55 (m, 8 H, -CH_2-CH_2-**CH_2**-**CH_2**-CH_2-CH_2-O-, -O-CH_2-CH_2-**CH_2**-**CH_2**-CH_3), 1.64 (tt, J=7.5, 7.2 Hz, 2 H, -Ar-CH_2-**CH_2**-CH_2-), 1.76 (tt, J=7.2, 6.5 Hz, -CH_2-**CH_2**-CH_2-O-Ar-, -Ar-O-CH_2-**CH_2**-CH_2-), 2.65 (t, *J*=7.5 Hz, 2 H, -Ar-**CH_2**-CH_2-), 3.98 - 4.16 (Two overlapping t, J=6.4 Hz, 4 H, -CH_2-**CH_2**-O-Ar-, -Ar-O-**CH_2**-CH_2-), 7.06 (d, *J*=8.9 Hz, 2 H, Ar), 7.12 (d, *J*=9.0 Hz, 2 H, Ar), 7.25 - 7.38 (m, 6 H, Ar), 7.67 (d, *J*=8.3 Hz, 2 H, Ar), 7.83 - 7.94 (m, 6 H, Ar), 8.08 (d, *J*=8.9 Hz, 2 H, Ar), 8.57 (s, 1 H, -**CH**=N-).
Elemental analysis: Calculated for $C_{44}H_{44}N_2O_4$: C, 79.49 %, H, 6.67 %, N, 4.21 %. Found: C, 79.47 %, H, 6.70 %, N, 4.08 %.

CB6OIBeO6 *- 4-[{[4-({6-[4-(4-Cyanophenyl)phenyl]hexyl}oxy)phenyl]methylidene}amino] phenyl 4-hexyloxybenzoate*:
Yield: 354 mg, 67.3 %.
Infrared $\bar{\nu}$ cm^{-1}: 2932, 2858 (sp^3 C-H); 2225 (C≡N); 1726 (C=O); 1604, 1573, 1511, 1494 (Ar C=C); 1247 (Ar C-O); 851, 835 (p. disubs. benzene C-H).
^{1}H NMR (300 MHz, DMSO-d_6) δ ppm: 0.89 (t, *J*=7.0 Hz, 3 H, -CH_2-**CH_3**), 1.27 - 1.52 (m, 10 H, -CH_2-CH_2-**CH_2**-**CH_2**-CH_2-CH_2-O-, -O-CH_2-CH_2-**CH_2**-**CH_2**-**CH_2**-CH_3), 1.64 (tt, *J*=7.3, 7.0 Hz, 2 H, -Ar-CH_2-**CH_2**-CH_2-), 1.75 (tt, *J*=7.0, 6.4 Hz, 4 H, -CH_2-**CH_2**-CH_2-O-Ar-, -Ar-O-CH_2-**CH_2**-CH_2-), 2.65 (t, *J*=7.4 Hz, 2 H, -Ar-**CH_2**-CH_2-), 4.01 - 4.13 (Two overlapping t, J=6.4 Hz, 4 H, -CH_2-**CH_2**-O-Ar-, -Ar-O-**CH_2**-CH_2-), 7.05 (d, *J*=8.7 Hz, 2 H, Ar), 7.11 (d, *J*=8.7 Hz, 2 H, Ar), 7.26 - 7.37 (m, 6 H, Ar), 7.66 (d, *J*=8.1 Hz, 2 H, Ar), 7.83 - 7.92 (m, 6 H, Ar), 8.08 (d, *J*=8.7 Hz, 2 H, Ar), 8.57 (s, 1 H, -**CH**=N-).
Elemental analysis: Calculated for $C_{45}H_{46}N_2O_4$: C, 79.62 %, H, 6.83 %, N, 4.13 %. Found: C, 79.63 %, H, 6.86 %, N, 3.98 %.

CB6OIBeO7 *- 4-[{[4-({6-[4-(4-Cyanophenyl)phenyl]hexyl}oxy)phenyl]methylidene}amino] phenyl 4-heptyloxybenzoate*:
Yield: 342 mg, 64.2 %.
Infrared $\bar{\nu}$ cm^{-1}: 3026 (sp^2 C-H); 2970, 2928, 2855 (sp^3 C-H); 2225 (C≡N); 1721 (C=O); 1603, 1572, 1511, 1494 (Ar C=C); 1253 (Ar C-O); 849, 826 (p. disubs. benzene C-H).
^{1}H NMR (300 MHz, DMSO-d_6) δ ppm: 0.88 (t, *J*=7.2 Hz, 3 H, -CH_2-**CH_3**), 1.23 - 1.55 (m, 12 H, -CH_2-CH_2-**CH_2**-**CH_2**-CH_2-CH_2-O-, -O-CH_2-CH_2-**CH_2**-**CH_2**-**CH_2**-**CH_2**-CH_3), 1.64 (tt, *J*=7.4, 7.2 Hz, 2 H, -Ar-CH_2-**CH_2**-CH_2-), 1.75 (tt, *J*=7.2, 6.4 Hz, 4 H, -CH_2-**CH_2**-CH_2-O-Ar-, -Ar-O-CH_2-**CH_2**-CH_2-), 2.65 (t, *J*=7.4 Hz, 2 H, -Ar-**CH_2**-CH_2-), 4.00 - 4.13 (Two overlapping t, J=6.4 Hz, 4 H, -CH_2-**CH_2**-O-Ar-, -Ar-O-**CH_2**-CH_2-), 7.06 (d, *J*=8.9 Hz, 2 H, Ar), 7.12 (d, *J*=9.0 Hz, 2 H, Ar), 7.24 - 7.38 (m, 6 H, Ar), 7.66 (d, *J*=8.3 Hz, 2 H, Ar), 7.82 - 7.94 (m, 6 H, Ar), 8.08 (d, *J*=8.9 Hz, 2 H, Ar), 8.57 (s, 1 H, -**CH**=N-).
Elemental analysis: Calculated for $C_{46}H_{48}N_2O_4$: C, 79.74 %, H, 6.98 %, N, 4.04 %. Found: C, 79.70 %, H, 6.95 %, N, 3.93 %.

CB6OIBeO8 *- 4-[{[4-({6-[4-(4-Cyanophenyl)phenyl]hexyl}oxy)phenyl]methylidene}amino] phenyl 4-octyloxybenzoate*:

Yield: 339 mg, 62.0 %.

Infrared $\bar{\nu}$ cm^{-1}: 2927, 2852 (sp^3 C-H); 2226 (C≡N); 1721 (C=O); 1604, 1572, 1512, 1494 (Ar C=C); 1252 (Ar C-O); 851, 827 (p. disubs. benzene C-H).

^{1}H NMR (300 MHz, DMSO-d_6) δ ppm: 0.87 (t, *J*=7.0 Hz, 3 H, -CH$_2$-**CH$_3$**), 1.22 - 1.55 (m, 14 H, -CH$_2$-CH$_2$-**CH$_2$**-**CH$_2$**-CH$_2$-CH$_2$-O-, -O-CH$_2$-CH$_2$-**CH$_2$**-**CH$_2$**-**CH$_2$**-**CH$_2$**-**CH$_2$**-CH$_3$), 1.64 (tt, *J*=7.6, 7.2 Hz, 2 H, -Ar-CH$_2$-**CH$_2$**-CH$_2$-), 1.75 (tt, *J*=7.2, 6.4 Hz, 4 H, -CH$_2$-**CH$_2$**-CH$_2$-O-Ar-, -Ar-O-CH$_2$-**CH$_2$**-CH$_2$-), 2.65 (t, *J*=7.6 Hz, 2 H, -Ar-**CH$_2$**-CH$_2$-), 4.01 - 4.13 (Two overlapping t, J=6.4 Hz, 4 H, -CH$_2$-**CH$_2$**-O-Ar-, -Ar-O-**CH$_2$**-CH$_2$-), 7.06 (d, *J*=8.9 Hz, 2 H, Ar), 7.12 (d, *J*=9.0 Hz, 2 H, Ar), 7.27 - 7.37 (m, 6 H, Ar), 7.67 (d, *J*=8.3 Hz, 2 H, Ar), 7.84 - 7.93 (m, 6 H, Ar), 8.08 (d, *J*=8.9 Hz, 2 H, Ar), 8.58 (s, 1 H, -**CH**=N-).

Elemental analysis: Calculated for $C_{47}H_{50}N_2O_4$: C, 79.86 %, H, 7.13 %, N, 3.96 %. Found: C, 79.96 %, H, 7.18 %, N, 3.81 %.

CB6OIBeO9 *- 4-[{[4-({6-[4-(4-Cyanophenyl)phenyl]hexyl}oxy)phenyl]methylidene}amino] phenyl 4-nonyloxybenzoate*:

Yield: 312 mg, 55.1 %.

Infrared $\bar{\nu}$ cm^{-1}: 2922, 2868, 2851 (sp^3 C-H); 2226 (C≡N); 1717 (C=O); 1602, 1572, 1507, 1495 (Ar C=C); 1246 (Ar C-O); 838, 821 (p. disubs. benzene C-H).

^{1}H NMR (300 MHz, DMSO-d_6) δ ppm: 0.87 (t, *J*=7.1 Hz, 3 H, -CH$_2$-**CH$_3$**), 1.22 - 1.51 (m, 16H, -CH$_2$-CH$_2$-**CH$_2$**-**CH$_2$**-CH$_2$-CH$_2$-O-, -O-CH$_2$-CH$_2$-**CH$_2$**-**CH$_2$**-**CH$_2$**-**CH$_2$**-**CH$_2$**-**CH$_2$**-CH$_3$), 1.64 (tt, *J*=7.4, 7.2 Hz, 2 H, -Ar-CH$_2$-**CH$_2$**-CH$_2$-), 1.75 (tt, *J*=7.2, 6.4 Hz, 4 H, -CH$_2$-**CH$_2$**-CH$_2$-O-Ar-, -Ar-O-CH$_2$-**CH$_2$**-CH$_2$-), 2.65 (t, *J*=7.4 Hz, 2 H, -Ar-**CH$_2$**-CH$_2$-), 4.00 - 4.14 (Two overlapping t, J=6.4 Hz, 4 H, -CH$_2$-**CH$_2$**-O-Ar-, -Ar-O-**CH$_2$**-CH$_2$-), 7.07 (d, *J*=8.9 Hz, 2 H, Ar), 7.12 (d, *J*=8.9 Hz, 2 H, Ar), 7.24 - 7.40 (m, 6 H, Ar), 7.67 (d, *J*=8.5 Hz, 2 H, Ar), 7.82 - 7.96 (m, 6 H, Ar), 8.08 (d, *J*=8.9 Hz, 2 H, Ar), 8.58 (s, 1 H, -**CH**=N-).

Elemental analysis: Calculated for $C_{48}H_{52}N_2O_4$: C, 79.97 %, H, 7.27 %, N, 3.89 %. Found: C, 79.98 %, H, 7.31 %, N, 3.73 %

CB6OIBeO10 *- 4-[{[4-({6-[4-(4-Cyanophenyl)phenyl]hexyl}oxy)phenyl]methylidene}amino] phenyl 4-heptyloxybenzoate*:

Yield: 304 mg, 53.6 %.

Infrared $\bar{\nu}$ cm^{-1}: 3036 (sp^2 C-H); 2936, 2917, 2848 (sp^3 C-H); 2225 (C≡N); 1718 (C=O); 1603, 1572, 1510, 1495 (Ar C=C); 1251 (Ar C-O); 844, 811 (p. disubs. benzene C-H).

^{1}H NMR (300 MHz, DMSO-d_6) δ ppm: 0.86 (t, *J*=7.0 Hz, 3 H, -CH$_2$-**CH$_3$**), 1.08 - 1.71 (m, 20H, -CH$_2$-**CH$_2$**-**CH$_2$**-**CH$_2$**-CH$_2$-CH$_2$-O-, -O-CH$_2$-CH$_2$-**CH$_2$**-**CH$_2$**-**CH$_2$**-**CH$_2$**-**CH$_2$**-**CH$_2$**-**CH$_2$**-CH$_3$), 1.75 (tt, *J*=7.2, 6.4 Hz, 4 H, -CH$_2$-**CH$_2$**-CH$_2$-O-Ar-, -Ar-O-CH$_2$-**CH$_2$**-CH$_2$-), 2.66 (t, *J*=7.0 Hz, 2 H, -Ar-**CH$_2$**-CH$_2$-), 4.01 - 4.13 (Two overlapping t, J=6.4 Hz, 4 H, -CH$_2$-**CH$_2$**-O-Ar-, -Ar-O-**CH$_2$**-CH$_2$-), 7.06 (d, *J*=8.9 Hz, 2 H, Ar), 7.12 (d, *J*=9.0 Hz, 2 H, Ar), 7.26 - 7.38 (m, 6 H, Ar), 7.67 (d, *J*=8.3 Hz, 2 H, Ar), 7.84 - 7.93 (m, 6 H, Ar), 8.08 (d, *J*=8.7 Hz, 2 H, Ar), 8.58 (s, 1 H, -**CH**=N-).

Elemental analysis: Calculated for $C_{49}H_{54}N_2O_4$: C, 80.08 %, H, 7.41 %, N, 3.81 %. Found: C, 80.25 %, H, 7.45 %, N, 3.66 %

Supplementary references

[1] E. Gorecka, N. Vaupotič, A. Zep, D. Pociecha, From Sponges to Nanotubes: A Change of Nanocrystal Morphology for Acute-Angle Bent-Core Molecules, *Angew. Chem. Int. Ed*., **55**, 12238 –12242 (2016).

[2] Ch. Dressel, T. Reppe, M. Prehm, M. Brautzsch, C. Tschierske, Chiral self-sorting and amplification in isotropic liquids of achiral molecules, *Nature Chem.*, **6**, 971-977 (2014).

[3] D. A. Paterson et al., Understanding the twist-bend nematic phase: the characterisation of 1-(4-cyanobiphenyl-4′-yloxy)-6-(4-cyanobiphenyl-4′-yl)hexane (CB6OCB) and comparison with CB7CB, *Soft Matter*, **12**, 6827 (2016).

[4] P. K. Freeman, J. S. Jang, N. Ramnath, The photochemistry of polyhaloarenes. 10. The photochemistry of 4-bromobiphenyl, *J. Org. Chem.*, **56**, 6072 (1991).

[5] S. M. Jansze, A. Martinez-Felipe, J. M. D. Storey, A. T. M. Marcelis, C. T. Imrie, A twist-bend nematic phase driven by hydrogen bonding, *Angew. Chem. Int. Ed.*, **54**, 643 (2015).

[6] D. Coates, G. W. Gray, Properties of the liquid crystals formed by some 4′-substituted 4-(β-p-substituted arylethyl)biphenyls, *J. Chem. Soc.-Perkin Trans.*, **2**, 863 (1976).

[7] M. K. Reddy et al., ^{13}C–^{1}H dipolar couplings for probing rod-like hydrogen bonded mesogens, *New J. Chem.*, **37,** 3195 (2013).
